# Bioactivity of Olive Oil Phenols in Neuroprotection

**DOI:** 10.3390/ijms18112230

**Published:** 2017-10-25

**Authors:** Cristina Angeloni, Marco Malaguti, Maria Cristina Barbalace, Silvana Hrelia

**Affiliations:** 1 School of Pharmacy, University of Camerino, 62032 Camerino, Italy; cristina.angeloni@unicam.it; 2 Department for Life Quality Studies, Alma Mater Studiorum-University of Bologna, 40126 Bologna, Italy; marco.malaguti@unibo.it (M.M); maria.barbalace2@unibo.it (M.C.B.)

**Keywords:** olive oil, tyrosol, hydroxytyrosol, oleuropein, oleocanthal, oxidative stress, neurodegeneration, Parkinson’s disease, Alzheimer’s disease, multiple sclerosis

## Abstract

Neurological disorders such as stroke, Alzheimer’s and Parkinson’s diseases are associated with high morbidity and mortality, and few or no effective options are available for their treatment. These disorders share common pathological characteristics like the induction of oxidative stress, abnormal protein aggregation, perturbed Ca^2+^ homeostasis, excitotoxicity, inflammation and apoptosis. A large body of evidence supports the beneficial effects of the Mediterranean diet in preventing neurodegeneration. As the Mediterranean diet is characterized by a high consumption of extra-virgin olive oil it has been hypothesized that olive oil, and in particular its phenols, could be responsible for the beneficial effect of the Mediterranean diet. This review provides an updated vision of the beneficial properties of olive oil and olive oil phenols in preventing/counteracting both acute and chronic neurodegenerative diseases.

## 1. Introduction

Neurodegenerative disorders, both chronic, such as Alzheimer’s disease (AD) and Parkinson’s disease (PD), and acute such as stroke and spinal cord injury (SCI), are associated with high morbidity and mortality, and few or no effective options are available for their treatment [1,2]. These diseases represent a primary health problem especially in the aging population [3]. For example, AD is the most common form of dementia and affects around 27 million people worldwide with incidence increasing from 1% between the ages of 60 and 70 to 6–8% by 85 years [4,5]. PD, the second most prevalent neurodegenerative disease, affects 1% to 2% of the population above the age of 65 [6,7]. In 2005, stroke was responsible for 5.7 million (16.6%) deaths [7] and SCI affects 1.3 million North Americans [8]. There is a strict correlation between acute and chronic brain diseases as acute brain injuries are risk factors associated with chronic neurodegenerative diseases such as AD and PD [9,10,11,12]. These disorders share common pathological characteristics such as the induction of oxidative stress, abnormal protein aggregation, perturbed Ca^2+^ homeostasis, excitotoxicity, inflammation and apoptosis [13,14]. In recent years, many studies have been focused on natural phytocomponents present in food as important bioactive molecules against age-related chronic diseases as neurodegenerative diseases [15,16,17,18,19,20] and a large body of evidence supports the beneficial effects of the Mediterranean diet (MD) in preventing neurodegeneration, possibly due to its richness in phenols [21,22,23,24,25]. Phytochemicals exert different biological activity, including antioxidant, antiallergic, anti-inflammatory, antiviral, antiproliferative, and anticarcinogenic effects [26,27,28]. MD is characterized by a daily consumption of extra-virgin olive oil in amounts ranging from 25 to 50 g/day [29,30,31], so it has been hypothesized that olive oil phenols could in part be responsible for the beneficial effect of the MD [32,33].

The olive tree (*Olea europaea*) synthesizes different phenols, found mainly in the leaves and drupes, used as defense against microbial and fungal invasion as well as to give leaves and drupes an unpleasant taste that discourages leaf-eating insects [34]. Natural phenols have been demonstrated to have many biological activities: they are able to modulate cell redox state [35] through direct action on enzymes, proteins, receptors, and different signaling pathways [36,37], as well as to interfere with biochemical homeostasis [38,39]. It has been shown that some of these effects are related to epigenetic modifications of the chromatin [40,41]. This review provides an updated vision of the beneficial properties of olive oil and olive oil phytochemicals (phenols and triterpenes) in preventing/counteracting both acute and chronic neurodegenerative diseases.

## 2. Methods

The Pubmed database was searched up to September 2017 using the keywords “Alzheimer’s disease”, “Parkinson’s disease”, “traumatic brain injury”, “spinal cord injury”, “stroke”, “amyotrophic lateral sclerosis”, “multiple sclerosis”, “ischemic brain injury”, “brain ipoxia”, “Huntington’s disease”, “oxidative stress”, “autophagy”, “proteasome” in combination with “olive oil”, “oleocanthal”, “tyrosol”, “hydroxytyrosol”, “oleuropein”. We also examined the reference lists of the retrieved articles. Criteria for inclusion in the review were as follows: (1) English language full-length publication in a peer-reviewed journal; (2) articles that directly addressed the topic of olive oil and its phenols. The ClinicalTrials.gov database was investigated using the same search criteria.

## 3. Olive Oil Phenols

**Figure 1:**
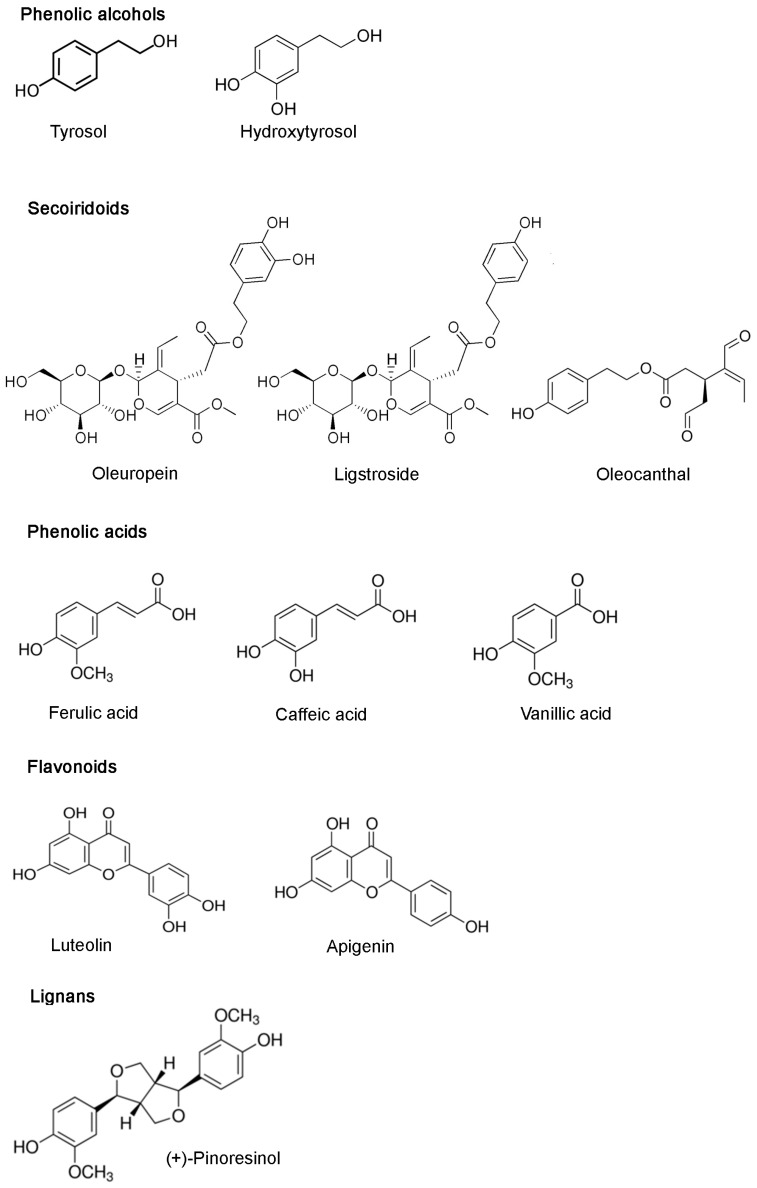
Main phenolic subclasses in olive oil and some representative compounds.

More than 200 different chemical compounds have been detected in olive oil including sterols, carotenoids, triterpenic alcohols and phenolic compounds. Phenolic compounds are also the main antioxidants found in virgin olive oil, which contains both hydrophilic and lipophilic phenols [42]. The main phenolic subclasses present in olive oil are phenolic alcohols, phenolic acids, flavonoids, lignans, and secoiridoids [43,44]. In Figure 1 some representative examples of the chemical structures of these compounds are reported.

Quantitatively, the class of secoiridoids is the most represented in olive oil. These chemicals derive from the cyclopentanopyran structural unit, known also as iridoid structure, after the breakage of the ciclopentane ring. The main secoiridoids in olive oil are oleuropein and ligstroside, their aglycones result constituted by elenolic acid esterified to hydroxytyrosol or tyrosol respectively [34]. Other secoiridoids, derived from oleuropein and ligstroside, are oleacein and oleocanthal, the dialdehydic form of decarboxymethyl elenolic acid bound to hydroxytyrosol or tyrosol respectively [34].

As a consequence of the oil production and aging processes, secoiridoids may easily hydrolyse raising the level of free hydroxytyrosol and tyrosol in oils [45].

Polyphenols content is responsible for the taste of different olive oils: hydroxytyrosol determines the bitter taste of olives and oils, while the stinging effect at pharynx level perceived after extra virgin olive oil ingestion is due to oleocanthal, as described by Beauchamp et al., in 2005 [46] who also demonstrated its ibuprofen-such as anti-inflammatory profile [46].

Many different studies tried to define the total phenolic content of olive oils. Of course this is a very hard duty, since many factors such as cultivars, climate conditions, ripening stage of the olives, but also olive oil production processes and storage affect olive phenolic content and composition [47]. Therefore, according to different studies, the amount of polyphenols in olive oil may range widely from 200 to 1000 mg/kg [34,45,48].

Since hydroxytyrosol is the main phenolic compound present in olive oil, some authors analyzed more than 250 extra virgin olive oil samples estimating that its median content is 137 mg/kg, while the median content of oleocanthal is 85 mg/kg [44]. Other authors tried to calculate the daily hydroxytyrosol intake concluding that it may not exceed 7 mg/day [42,45]. Globally, Dilis and Trichopoulou reported a calculated daily intake of phenolic compounds of about 17 mg [49]. De La Torre estimated for a 25–50 mL olive oil intake/day a 9 mg polyphenol intake, where at least 1 mg was due to hydroxytyrosol and tyrosol, and 8 mg represented by the oleuropein- and ligstroside-aglycons [50].

### Olive Oil Phenols Bioavailability

After olive oil consumption its polyphenols are quickly metabolized and absorbed. Through the gastro intestinal tract, secoiridoid aglycones such as oleuropein and ligstroside are mainly hydrolyzed in elenolic acid plus hydroxytyrosol and tyrosol respectively [51]. Hydroxytyrosol and tyrosol are absorbed in a dose-dependent manner [52,53], their peak plasma levels are found 1 h after ingestion [53,54], while peak urine concentrations are detected 0–2 h after assumption [55,56]. However, when administered as aqueous solution their bioavailability is particularly low, highlighting how the vehicle is important to determine hydroxytyrosol and tyrosol bioavailability [57].

Once intravenously injected in rats, ^14^C labeled-hydroxytyrosol quickly disappeared from plasma due to its fast metabolism and distribution to different tissues [58]. Hydroxytyrosol metabolism is so fast that already 5 min after injection its metabolites are detectable in plasma, and its half-life has been estimated between 1 and 2 min. Therefore, despite their good absorption, hydroxytyrosol and tyrosol bioavailability result low due to the quick metabolism. Once absorbed, in fact, they undergo an extensive first passage metabolism and phase I/II biotransformation at intestinal and liver level. Ninety-eight percent of them are found in plasma and urine in a conjugated form, mainly glucurono-conjugated or, in less extent, sulfated [56]. Considering this strong metabolism, some authors proposed that hydroxytyrosol and tyrosol biological activity might be due to their metabolites. Some data, in fact, suggest that hydroxytyrosol-3-*O*-glucuronide has got a stronger antioxidant activity than hydroxytyrosol itself [59].

The blood brain barrier (BBB) has an extremely selective permeability. The aglycones of polyphenols can cross membranes by a passive diffusion mechanism and therefore can be better absorbed then their glycated counterparts [60].

Oleuropein aglycone can cross the BBB; in fact it has been detected in the rat brain parenchyma after administration of a phenolic extract from olive cake [61].

Hydroxytyrosol ^14^C radioactivity measured in different tissues showed that it is distributed to skeletal muscles, liver, heart, kidney, lung and brain, demonstrating that it can cross the BBB [58], an essential feature to explain its neuroprotective role. Interestingly, hydroxytyrosol plasma concentration does not only derive from olive oil polyphenols absorption, but also from dopamine degradation. In fact, hydroxytyrosol is a by-product of dopamine oxidative metabolism, a pathway that involves multiple enzymes, monoaminooxidase, aldehyde dehydrogenase and aldehyde reductase [50].

Oleocanthal bioavailability and its ability to cross the BBB have not yet fully established. It has been reported that a significant amount of human urine metabolites following 50 mL olive oil ingestion were from oleocanthal [62], providing evidence of the absorption and metabolism in the human body, however further studies are necessary to fully understand the metabolism and bioavailability of this compound.

## 4. Olive Oil Phenols and Oxidative Stress

Brain tissue, due to its high energy requirements, is characterized by high oxygen consumption and metabolic rate. Moreover, cells of brain tissue present a very high polyunsaturated fatty acid (PUFA) content and are known to have low antioxidant defenses. All together high oxygen consumption, high PUFA content and low antioxidant defenses make brain prone to oxidative stress [63,64,65]. Oxidative stress consists of a disequilibrium between free radical production and scavenging that leads to an abnormal generation of reactive oxygen species (ROS) and oxidative damage. It has been proposed and demonstrated that different neurodegenerative diseases share oxidative stress as common characteristic [66,67].

Many physiological and pathological conditions are potential source of reactive oxygen species (ROS) and are responsible for oxidative stress in brains affected by neurodegenerative diseases. Trace elements such as copper, iron, aluminum, mercury and arsenic enhance free radical generation [68,69]. Neuroinflammation typically occurs during the development and progression of AD, PD and other neurodegenerative diseases, and causes a strong increase of ROS level and induces oxidative stress [70,71,72,73]. Formation of superoxide anion and production of hydrogen peroxide are triggered by the induction of NADPH oxidase (NOX) subunit [74], this condition together with high nitric oxide level, produced by the induction of inducible nitric oxide synthase (iNOS) [75], results in the formation of peroxynitrite and nitrative stress [76]. Mitochondria are an additional potential site of ROS production. Oxidative stress induced by impaired mitochondrial functions has been reported in numerous neurodegenerative disorders such as multiple sclerosis (MS), PD, amyotrophic lateral sclerosis (ALS) and AD [77,78,79,80,81].

To date many reports demonstrated that phenolics from *Olea europaea* L., found also in extra virgin olive oil, exert strong antioxidant properties and are able to counteract oxidative stress in brain tissue. Oleuropein and hydroxytyrosol act as direct free radical scavengers, hydroxytyrosol and oleocanthal are strong cyclooxygenases (COX) inhibitors and oleuropein counteracts low density lipoprotein (LDL) oxidations [46,82,83,84,85].

Early evidence showed that hydroxytyrosol counteracts Fe^2+^- and NO-induced loss of cellular ATP and depolarization of mitochondrial membrane potential in murine dissociated brain cells [86].

More recently, long-term polyphenols-rich extra virgin olive oil dietary administration in mice counteracted age-related dysfunctions in motor coordination and improved oxidative stress biomarkers such as thiobarbituric acid reactive substances (TBARS) at cortex level. Moreover, it increases glutathione peroxidase (GPx) activity in some brain regions such as cortex and cerebellum [29].

Recently, some authors evaluated oleuropein ability to counteract arsenic toxicity in mice. Arsenic administered at 5 mg/kg/die through drinking water induces oxidative stress in multiple tissues such as kidney, liver and brain as demonstrated by accumulation of oxidative damage biomarkers, protein carbonyls, malondialdehyde (MDA) and depletion of antioxidant defenses and NO [87]. Oleuropein treatment (30 mg/kg/die for 15 days) partially ameliorated arsenic-induced oxidative stress and NO production [88]. Soni et al. [89] explored hydroxytyrosol protective effect on arsenic-induced oxidative stress and mitochondrial dysfunction in rat brains and obtained similar results. They demonstrated that hydroxytyrosol (10 mg/kg/die for 8 weeks) counteracted arsenic depletion of catalase (CAT), manganese superoxide dismutase (MnSOD), and the reduction of mitochondrial complexes I, II, IV activities, concluding that hydroxytyrosol might be considered a potential mitoprotective agent. 

Olive oil administered to rats subjected to brain hypoxia–reoxygenation was demonstrated to exert antioxidant and cytoprotective activity decreasing brain cell death, lipid peroxide level, counteracting the decrease in glutathione levels and inhibiting prostaglandin E2 (PGE2) overproduction in brain tissues [90].

Recently some evidence is arising showing that both hydroxytyrosol and oleuropein antioxidant effects in the brain are mediated by the activation of the Keap1-Nrf2 pathway, which downstream up-regulates cytoprotective enzymes such as thioredoxin reductase, heme oxygenase 1 (HO-1), NAD(P)H:quinone oxidoreductase 1 (NQO1) and glutamate–cysteine ligase [91,92]. Moreover, even though it acts also as direct scavenger, hydroxytyrosol neuroprotective activity vanish when Nrf2 is knocked down [91]. In fact, it has been demonstrated that intracellular phenolics concentrations in neuronal cells is in the nanomolar-low micromolar range, not sufficient for a direct antioxidant effect but high enough to activate a hormetic dose-response by modulating intracellular signaling pathways [93].

## 5. Olive Oil Phenols in Counteracting Loss of Proteostasis

Many neurodegenerative diseases such as AD, PD, Lewy body dementia, Pick disease, frontotemporal dementia, Huntington’s disease (HD), and ALS are associated with perturbed proteostasis [94]. The proteostasis network includes different pathways related to protein synthesis, folding, trafficking, secretion, and degradation distributed in different compartments inside the cell. Dysfunctional proteins are safely degraded via the ubiquitin–proteasome system and the autophagy pathway [95]. 

In recent years, the role of autophagy impairment in neurodegenerative disease has been widely demonstrated [96,97]. Autophagy maintains cellular homeostasis through removal and recycling of damaged macromolecules and organelles [98]. The autophagy pathway implies sequestration of cytoplasmic components in double-membrane vesicles termed autophagosomes that subsequently fuse with lysosomes to form autophagolysosomes [99]. Autophagy is triggered by different stimuli: starvation, the presence of deposited materials and aged cellular organelles, principally mitochondria (mitophagy) [100]. The delivery of cytoplasmic proteins to the lysosomes by autophagy can follow different pathways: CMA (chaperone-mediated autophagy), macroautophagy and microautophagy [101]. 

Macroautophagy initiation is under the control of ULK1 that is negatively regulated by mammalian target of rapamycin (mTOR) by phosphorylation [102]. Rigacci et al. [100] investigated the molecular and cellular mechanisms of macroautophagy induction by oleuropein aglycone using cultured neuroblastoma cells and an oleuropein aglycone fed mouse model of amyloid beta (Aβ) deposition. Oleuropein aglycone induced autophagy in cultured cells through the Ca^2+^-calmodulin-dependent kinase β-AMPK axis. The correlation between AMPK activation and mTOR inhibition was demonstrated in the oleuropein aglycone-fed animal model in which decreased phospho-mTOR immunoreactivity and phosphorylated mTOR substrate p70 S6K levels matched enhanced phospho-AMPK levels, supporting the idea that autophagy activation by oleuropein aglycone proceeds through mTOR inhibition.

A subsequent study evaluated the effect of picomolar doses of oleuropein on the modulation of autophagy in nerve growth factor (NGF)-differentiated PC12 cells exposed to the potent parkinsonian toxin 6-hydroxydopamine (6-OHDA) [103]. Interestingly, the results of this study were in contrast with the results obtained by Rigacci et al., in fact oleuropein did not favor LC3-II accumulation and increased the protein level of p62, an indicator of inhibition of autophagy, suggesting that picomolar doses of oleuropein inhibit, rather than increase autophagy. In agreement with these results, the study of Olivan et al. [104] investigated the protective effect of extra virgin olive oil nutritional supplementation in an ALS mouse model. They observed that olive oil induced a significantly down-regulation of the expression of LC3 and Beclin1 genes in muscles together with an increased survival rate, improved motor coordination and reduced muscle damage. 

In conclusion, the effect of oleuropein on autophagy depends on the dose as micromolar doses increase autophagy, meanwhile picolomolar ones inhibit this degradative pathway. Of course further studies should be carried out to better understand the role of nutritional doses of olive oil in the modulation of the autophagic flux.

The proteasome, a nonlysosomal threonine protease, is a complex present in the cytoplasm of cells [95] and responsible for the turnover of both normal and damaged intracellular proteins [105]. Most proteins degraded by proteasomes are tagged by polyubiquitination [106]. These proteins are then conveyed into the core of the proteasome where they are degraded into short peptides by the internal protease activities [101]. The impairment of the ubiquitin-proteasome system has been implicated in the pathogenesis of a wide variety of neurodegenerative disorders [107] and its induction represents an emerging therapeutic target to counteract these diseases. To our best knowledge, only one study investigated the effect of oleuropein on the proteasome of human embryonic fibroblasts and showed that this olive oil component is able to enhance proteasome activities in vitro stronger than other known chemical activators, possibly through conformational changes of the proteasome [108]. In conclusion, the effects of olive oil and olive oil components on autophagy are inconsistent, meanwhile the modulation of the proteasome by olive oil has been investigated only on fibroblast suggesting that these aspects should be further clarified.

## 6. Protective Effects of Olive Oil and Olive Oil Phenols against Acute Neurodegeneration

### 6.1. Ischemic Brain Injury 

Stroke is a complex neurodegenerative disorder characterized by interruption of blood flow to brain resulting in tissue hypoxia. Increasing evidence suggests that cerebral hypoxia and particularly reperfusion are responsible for the release of excitatory amino acids, with subsequent receptor activation leading to calcium influx, metabolic and electrophysiological dysfunction, lipid peroxidation and oxidative stress [109]. As ischemic events are increased in elderly individuals [110], they are particularly prone to the deleterious effects of these events. On these basis, the antioxidant and anti-inflammatory olive oil phenols have been considered as neuroprotective nutraceuticals to be used in ischemic brain injury prevention and therapy. 

The ability of virgin olive oil to counteract hypoxic brain damage was evaluated in a hypoxia-reoxygenation in vitro model with fresh brain slices of rats receiving virgin olive oil for 30 days [90]. Virgin olive oil counteracted cell death by reducing lipid peroxidation, brain prostaglandin E2, and NO production and by increasing glutathione concentration. In an attempt to better clarify the contribution of olive oil phenols to the protection against hypoxia-reoxygenation, the same authors investigated the possible neuroprotective effect of hydroxytyrosol and hydroxytyrosol acetate in the same experimental model of hypoxia–reoxygenation [111]. Hydroxytyrosol and hydroxytyrosol acetate inhibited LDH efflux in a concentration-dependent manner and interestingly, other well-known antioxidants such as vitamin E and *N*-acetyl-cysteine, had no neuroprotective effect in this experimental model suggesting that hydroxytyrosol and hydroxytyrosol acetate could have additional activities besides the antioxidant one against hypoxia-reoxygenation. 

Different in vivo studies evaluated the effect of virgin olive oil and its phenols in different experimental model of ischemia/reperfusion in rodents.

Mohagheghi et al. [112] investigated the impact of dietary virgin olive oil on brain infarct volume, brain edema, BBB permeability, and neurological dysfunction resulting from transient middle cerebral artery occlusion (MCAO) in rats receiving 0.25–0.75 mL/kg virgin olive oil for 30 days. The pretreatment with dietary virgin olive oil reduced infarct volume, brain edema, BBB permeability, and neurobehavioral deficit scores. Another study investigated the protective effect of olive oil against ischemia/reperfusion in mice. Mice were treated with olive oil for a week, then, ischemia was induced by common carotid artery ligation and a week after ischemia the mice were post-treated with olive oil. The treatment decreased cell death in the hippocampus CA1 suggesting a protective role of olive oil against ischemia/reperfusion injury in this specific brain area. Moreover, Rabiei et al. [113] observed that virgin olive oil administered to rats for 30 days influenced brain lipidomics during MCAO. In particular, virgin olive oil increased brain phosphatidylcholine, cholesterol ester and cholesterol, triglyceride, and cerebroside levels and decreased the brain ceramide levels indicating that the olive oil could partly exert its effects via change in brain lipid levels.

In another study, the effects of tyrosol on infarct volume and sensory motor function deficit after MCAO was investigated in rats [114]. Tyrosol showed a dose-dependent neuroprotective effect that peaked at 64.9% in rats treated with 30 mg/kg of tyrosol. In rotarod, beam balance, and foot fault tests, tyrosol exhibited protective effects against the sensory motor dysfunction. 

Oleuropein treatment provides neuroprotective activity by decreasing cerebral infarct volume and improving neurobehavioral functions after cerebral I/R injury in mice [115]. Moreover, oleuropein showed an anti-apoptotic effect by increasing the ratio of Bcl-2/Bax through the up-regulation of the expression of Bcl-2 and the down-regulation of the expression of Bax.

Of note, a study evaluating the association between the traditional MD and the incidence of mortality from cerebrovascular disease (CBVD), showed that increased adherence to the MD was inversely associated with CBVD incidence (adjusted hazard ratio = 0.85, 95% confidence interval: 0.74, 0.96) and mortality (adjusted hazard ratio = 0.88, 95% CI: 0.73, 1.06) [116]. These inverse trends were mostly evident among women and with respect to ischemic rather than hemorrhagic CBVD and were largely driven by consumption of vegetables, legumes, and olive oil. 

### 6.2. Spinal Cord Injury

SCI is a highly debilitating pathology [117] which is caused initially by traumatic mechanical injury (the primary injury) to the spinal cord that leads to the death of a number of neurons that cannot be recovered and regenerated. Unfortunately, neurons continue to die for hours following the trauma [118] and the outcome of SCI is closely related to the extent of the “secondary injury” mediated by a series of cellular, molecular and biochemical cascades including calcium ion influx, oxidative stress, inflammation, autoimmune response, vascular events, and apoptosis [119]. As the secondary injury appears to be susceptible to pharmacological interventions including the use of antioxidant and anti-inflammatory agents, many studies have been focused on the modulation of the mechanisms related to secondary injury.

Post-traumatic inflammation is characterized in part by the accumulation of neutrophils that play an important role in the pathogenesis of secondary injury by the release of inflammatory mediators [120]. Tissue myeloperoxidase (MPO) activity is a well-known biomarker of the extent of post-straumatic neutrophil infiltration [121]. Focusing on this aspect, the anti-inflammatory effect of oleuropein after spinal cord experimental injury was investigated in rats treated with a 20 mg/kg single dose of oleuropein (i.p). immediately or 1 h after trauma [122]. Oleuropein strongly decreased MPO activity, and interestingly the treatment at 1 h after the trauma was significantly more effective that the other. In the same experimental conditions, oleuropein treatment was able to counteract inflammation reducing the expression of tumor necrosis factor (TNF)-α, interleukin-1β (IL-1β), iNOS and COX-2 [123] and lipid peroxidation, reducing MDA, increasing reduced glutathione (GSH) level, attenuating myelin degradation in the dorsal funiculus, and counteracting apoptosis [124]. Impellizzeri et al. [123] investigated the effect of oleuropein aglycone, on the inflammatory response in a mouse model of spinal cord trauma. Oleuropein aglycone was administered in mice 1 and 6 h after trauma. The treatment with oleuropein aglycone significantly decreased histological damage, motor recovery, nuclear factor kappa B (NF-κB) expression and inhibitor kappa B (IκB-α) degradation, protein kinase A activity and expression, proinflammatory cytokines production such as TNF-α and IL-1β, IL-6 inducible iNOS expression, neutrophil infiltration, lipid peroxidation, nitrotyrosine and poly-ADP-ribose formation, glial cell-derived neurotrophic factor levels, and apoptosis. 

In conclusion, both oleuropein and its aglycon form are promising agents in counteracting the onset of inflammation and oxidative stress following SCI. 

## 7. Protective Effects of Olive Oil Phenols against Chronic Neurodegeneration

### 7.1. Alzheimer’s Disease

AD is the most common form of dementia in the elderly that afflicts about 30 million patients globally and is expected to increase enormously by 2050 [125]. Neuropathologically, AD is characterized by increased accumulation of intracellular neurofibrillary tangles (NFTs) of hyperphosphorylated tau protein and of extracellular Aβ protein deposits (Aβ plaques) derived from amyloid precursor protein (APP) cleavage by γ-secretase and β-secretase [126,127,128]. There are two different form of Alzheimer’s disease: the hereditary “familial” form is characterized by an early onset (before age 50) associated with mutations in the APP gene and the genes for PS1 or PS2. On the other hand, the second form of AD, known as sporadic or late-onset, affects the highest percentage of the population [129]. Although Aβ plaques and NFTs are the main hallmarks of AD, mitochondrial dysfunction, loss of calcium regulation, oxidative damage, and inflammation play an important role in the onset and progression of AD [128,130,131,132,133]. Nowadays, there are no effective therapies for AD and the only treatments available just alleviate disease symptoms. A large body of evidence supports the beneficial effects of the MD in attenuating AD-like pathology, mild cognitive impairment and its conversion to AD [130,134]. In particular, it has been suggested that olive oil phenolic components are key factors in improving aging- and disease-associated behavioral deficits [21,135,136].

Extra-virgin olive oil rich in phenols (total phenol dose/day, 6 mg/kg) administered chronically in the second half of mouse life span, improved contextual memory in the step-down test to levels similar to young animals and prevented the age-related impairment in motor coordination in the rotarod test [29]. This motor effect was correlated with reduced lipid peroxidation in the cerebellum. Another study examined the effects of extra virgin olive oil on learning and memory in SAMP8 mice, an age-related learning/memory impairment model associated with increased Aβ protein and brain oxidative damage [137]. Mice receiving olive oil had improved acquisition in the T-maze and spent more time with the novel object in one-trial novel object recognition versus mice which received coconut oil or butter. Moreover, olive oil increased brain glutathione levels suggesting reduced oxidative stress as a possible protective mechanism. These effects, plus increased glutathione reductase and SOD activity, and decreased tissue levels of 4-hydroxynoneal (4-HNE) and 3-nitrotyrosine were enhanced by extra virgin olive oil enriched with phenols. A recent investigation explored the effect of an extra virgin olive olive-enriched diet administered before or after the presence of Aβ deposit in TgSwDI mice [137]. Animals fed with olive oil for 6 months and before the starting of Aβ accumulation showed reduced Aβ and tau brain levels and a significant improvement in mouse cognitive behavior. These effects were associated to an enhanced Aβ clearance pathways and reduced brain production of Aβ via modulation of Aβ precursor protein processing. On the other hand, olive oil administrated after Aβ accumulation starts, showed improved clearance across the BBB and significant reduction in Aβ levels, but it did not affect tau levels or improved cognitive functions of TgSwDI mice suggesting that the long-term consumption of olive oil starting at early age is more effective in protecting against AD.

A number of different studies investigated the effect of single olive oil phenols both in vitro and in vivo. In vitro studies showed that oleuropein interferes with amyloid aggregation of amylin and Aβ42 skipping the formation of toxic oligomeric species [32,138]. Using transgenic *C. elegans* strains expressing Aβ42 as a simplified invertebrate model of AD, Diomede et al. [139] evidenced that worms grown on oleuropein supplemented medium were protected against plaque deposits, Aβ oligomer appearance. Moreover, worms did not evidence impairment of motility and displayed increased survival. Dietary supplementation of oleuropein to TgCRND8 mice had a protective effect by reducing de novo deposition of Aβ42 and promoting preformed plaque disassembly in the brain of young/middle-aged animals [39]. 

Pyroglutamate-modified Aβ peptides at amino acid position three (pE3-Aβ) are receiving great attention as possible key players in the pathogenesis of AD. pE3-Aβ is abundant in AD brain and has a high aggregation propensity, stability and cellular toxicity. In aged mice displaying increased pE3-Aβ in the brain deposits, oleuropein counteracted glutaminylcyclase-catalyzed pE3-Aβ production by reducing enzyme expression and interfered with both Aβ42 and pE3-Aβ aggregation [39,140]. Interestingly, the beneficial effects of oleuropein were dose-related and a mix of olive phenols resulted in quantitatively and qualitatively similar effects as those recorded with pure oleuropein [141], suggesting that the observed effects are not closely related to oleuropein by itself. The same Authors demonstrated that when Aβ42 is aggregated in the presence of oleuropein it is not toxic to neurons when injected into the rat nucleus basalis magnocellularis, which is homologous to the nucleus of Meynert in humans, and glia reactivity is reduced [142]. Oleuropein counteracted cognitive dysfunction induced by colchicine in the hippocampal CA1 area in rats improving the retention performance of the spatial navigation task in Morris water maze and attenuated colchicine induced oxidative stress reducing lipid peroxidation, nitrite level, caspase 3 activity and increasing CAT and GPx activities [143].

The potential protective effect of tyrosol and hydroxytyrosol against Aβ-induced toxicity has been investigated in cultured neuroblastoma N2a cells [144]. Both tyrosol and hydroxytyrosol reduced cell death induced by Aβ, even if the treatment with both of them was not able to prevent GSH decrease induced by H_2_O_2_ or Aβ. Interestingly, the presence of tyrosol and hydroxytyrosol attenuated the nuclear translocation of the NF-κB subunit induced by Aβ exposure, suggesting that these compounds act with an anti-inflammatory mechanism rather than an antioxidant one. The effect of hydroxytyrosol was also explored in vivo feeding APP/PS1 transgenic mice, a familial AD mouse model, with 5 mg/kg/day of hydroxytyrosol for 6 months [145]. Hydroxytyrosol did not attenuate brain Aβ accumulation in AD mice even if improved EEG alteration, reduced inflammation and mitochondrial oxidative stress by decreasing protein oxidation and the lipid peroxidation product 4- HNE. In addition, hydroxytyrosol increased SOD2 expression and GSH levels, meanwhile down-regulated HO-1 and NQO1 expression that were overexpressed in AD mice. It has recently evidenced that diabetes is a risk factor for AD onset and development, being insulin resistance the main link between diabetes and AD. In this context, Crespo et al. [146] demonstrated that hydroxytyrosol exerts beneficial effects on insulin resistance associated with AD in an astrocytic model of AD. Pre- and post-treatment with hydroxytyrosol counteracted cell death induced by Aβ(25–35) through the activation of Akt. In addition, hydroxytyrosol prevented the pronounced activation of mTOR, thereby restoring proper insulin signaling.

Oleocanthal has been investigated for its anti-aggregation activities on tau protein. Tau is a microtubule-associated protein that promotes microtubule assembly and stability. In Alzheimer’s disease, tau aggregates into NFTs. In vitro, oleocanthal inhibits the polymerization of tau protein through a covalent mechanism [147,148]. This aspect was first investigated by Li et al. [147] that showed that oleocanthal forms an adduct with the lysine residue corresponding to K311 in tau protein, which is a critical site for tau fibrillization, via initial Schiff base formation and thereby inhibiting tau fibrillization. This study has some limitations as it was carried out using the fibrillogenic short hexapeptide PHF6 which cannot be representative of a complex protein system and it is not fully adequate to deduce the exact mechanism of action of oleocanthal. Monti et al. [148] demonstrated that oleocanthal is capable of altering the fibrillization of tau protein reacting with the lysine ε-amino groups of the tau fragment K18 in an unspecific fashion. Subsequently, the same authors investigated the recognition process and the reaction profile between oleocanthal and the wild-type tau protein demonstrating that oleocanthal interact with tau-441, inducing stable conformational modifications of the protein secondary structure and also interfering with tau aggregation [149]. 

Pitt et al. [33] were the first to speculate that oleocanthal could alter Aβ aggregation states. In particular, analysis of oligomers in the presence of oleocanthal showed an upward shift in molecular weight and a decreased binding to synapses that was accompanied by significantly less synaptic deterioration. Moreover, oleocanthal treatment improved antibody clearance of oligomers. These results were further expanded by Abuznait et al. [38] that demonstrated both in cultured mice brain endothelial cells and in C57BL/6 wild-type mice that oleocanthal enhances Aβ clearance from the brain via up-regulation of P-glycoprotein (P-gp) and LDL receptor related protein-1 (LRP1), major Aβ transport proteins at the BBB. In addition, the potential modifications in Aβ degradation induced by oleocanthal were also studied by analyzing the levels of two enzymes implicated in the process: insulin degrading enzyme and neprilysin. Interestingly, oleocanthal up-regulated both of them. As these previous studies were not carried out using an AD model, the same research group investigated the effect of oleocanthal on Aβ load in the brain parenchyma in TgSwDI mice, a well-known mouse model of AD, and on Aβ deposit on brain microvessels. A 4-week treatment with oleocanthal reduced amyloid load in the hippocampal parenchyma and microvessels. This reduction was associated with enhanced cerebral clearance of Aβ across the BBB increasing the expression of P-gp and LRP1, and activation of the apolipoprotein E (ApoE)-dependent amyloid clearance pathway in the mice brains by increasing the expression of ATP-binding cassette transporter 1 (ABCA1), ApoE, and the nuclear receptors PPARγ. Oleocanthal was also able to counteract inflammation in the brains reducing astrocytes activation and IL-1β levels [31]. Very recently, the same experimental group showed that oleocanthal attenuated the inflammation induced by Aβ oligomer in the human brain astrocytoma cell culture CCF-STTG1 reducing the expression of IL-6 and glial fibrillary acidic protein (GFAP), and restoring astrocyte neuro-supportive function by preventing Aβ oligomer down-regulation effects on glutamine transporter (GLT1) and glucose transporter (GLUT1) [150]. Moreover, oleocanthal counteracted the down regulation of the synaptic proteins GLT1 and PSD-95 induced by Aβ oligomer in SH-SY5Y cell line transfected with APP695. On the basis of these results, the Authors suggested that the effect of oleocanthal on neuronal cells could be direct and not mediated by astrocyte protective crosstalk.

As previously reported, oleocanthal has Ibuprofen-like activity thanks to its ability to inhibits COX-1 and COX-2 [46] and this strong anti-inflammatory activity makes oleocanthal a promising compound in counteracting AD-associated neuroinflammation.

**Figure 2:**
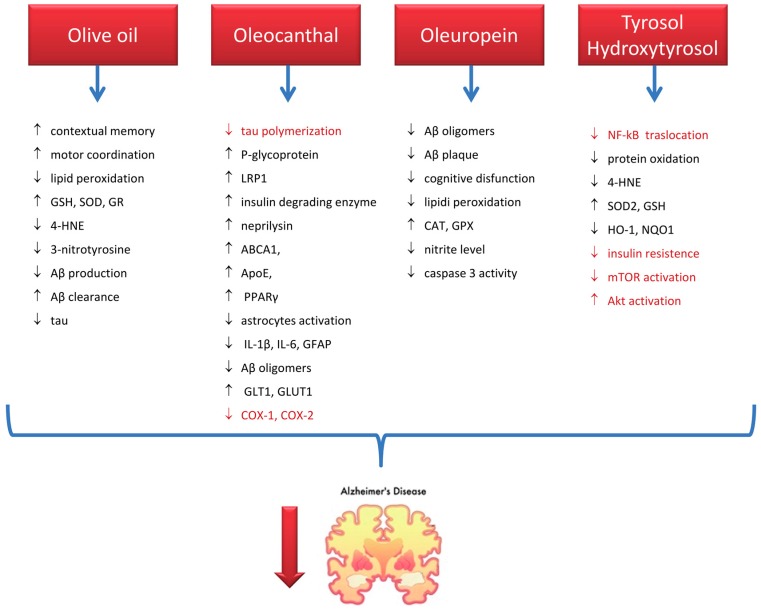
Mechanisms of action of olive oil and its phenols in preventing/counteracting AD. Black indicates results from animal studies, red from in vitro studies. ↓ stands for inhibition, ↑ stands for activation. GSH: reduced glutathione, SOD: superoxide dismutase, GR: glutathione reductase; 4:HNE: 4-hydroxynoneal, Aβ: amyloid beta, LRP1: LDL receptor related protein-1, ABCA1: ATP-binding cassette transporter 1, APOE: Apolipoprotein E, PPARγ: peroxisome proliferator–activated receptor gamma, IL: interleukin, GFAP: glial fibrillary acidic protein, GLT1: glutamine transporter 1, GLUT1: glucose transporter 1, COX: cyclooxygenase, CAT: catalase, GPX: glutathione peroxidase, HO-1: heme oxygenase 1, NQO1: NAD(P)H:quinone oxidoreductase 1, mTOR: mammalian target of rapamycin.

Collectively these results (Figure 2) evidence that olive oil and its phenolic components play a beneficial effect in AD via targeting multiple pathological aspects of this disease and confirm the hypothesis that olive oil consumption is strongly associated with the positive effect showed by MD on AD risk. [134,151]

### 7.2. Parkinson’s Disease

Parkinson’s disease is characterized by the progressive loss of dopaminergic neurons in the midbrain region known as *substantia nigra pars compacta* and by the presence of cytoplasmic protein aggregates, called Lewy body, and Lewy neurites in remaining neurons [152,153]. Although the precise etiology of PD remains unknown, it is becoming increasingly clear that the onset of PD is multi-factorial and involves disruptions in multiple cellular systems. In particular, the loss of dopaminergic neurons has been associated to different causes including mitochondrial dysfunction, oxidative stress, loss of glutathione, neuroinflammation, loss of neurotrophic factor signaling, abnormal protein accumulation, and environmental toxins [154,155,156]. The highest number of PD cases are sporadic [157,158], and only 10% are of genetic origin [159], mainly linked to mutations in α-synuclein protein [160], a principal component of Lewy body inclusions [161], parkin [159,162], PTEN-induced putative kinase 1 (PINK1), dardarin, and protein deglycase (DJ-1) [163]. 

Dopaminergic neurons are highly prone to oxidative stress as dopamine itself can spontaneously undergo auto-oxidation leading to the production of toxic molecules such as hydrogen peroxide, superoxide radicals and dopamine (DA)-quinone species [47,164]. Oxidized DA can reacts with sulfhydryl groups in cysteinyl proteins to form Michael adduct and generates ROS through redox cycling [165,166,167]. In addition, mitochondrial dysfunction and neuroinflammation seems to play a fundamental role in increasing ROS level in the substantia nigra of PD patients [168].

Current therapies for PD are not able to prevent dopaminergic neuron loss or stop the progression of the disease, only delay the onset or reduce the motor symptoms. The gold standard therapy against PD relies on restoring the optimum level of DA and its associated signaling pathways by the administration of l-3,4-dihydroxyphenylalanine (l-DOPA), a precursor of DA [169]. Another strategy for PD therapy is the use of monoamine oxidase (MAO)-B inhibitors in order to stop DA degradation [170]. Both of these therapeutic approach have important side effects and, in this milieu, different strategies are being under investigation [171]. Among them, nutraceutical approaches involving natural compounds present in common food such as olive oil has been shown to impart beneficial effect in PD [17,172,173,174,175]. 

To our knowledge, no studies have been carried out to explore the effect of olive oil in counteracting Parkinson’s disease, on the other hand, many studies have investigated the role of single olive oil phenols in modulating the cellular system alteration involved in the onset of the disease.

Dewapriya et al. [176] examined the protective effect of tyrosol against the parkinsonian toxin1-methyl-4-phenylpyridinium (MPP+) in dopaminergic CATH.a neurons. MPP+ selectively enters into DA-producing neurons and inhibits the mitochondrial electron transporter chain leading to oxidative stress, which ultimately causes neuronal death [177]. Tyrosol attenuated mitochondrial dysfunction and intracellular ATP depletion induced by MPP+. Moreover, tyrosol up-regulated the expression of the antioxidant enzymes SOD-1 and SOD-2 and DJ-1 and increased the activation of Akt, suggesting that tyrosol, in this specific experimental conditions, achieved neuroprotection probably via an Akt-signaling-pathway-dependent mechanism.

In another in vitro model of Parkinson’s disease, oleuropein (20 and 25 µg/mL) decreased cell damage and reduced oxidative stress and apoptosis induced by 6-OHDA in PC12 cells [178]. This data were further confirmed by a recent study evaluating the neuroprotective effect of picomolar doses of oleuropein in PC12 cells exposed to 6-OHDA [103]. In particular, oleuropein lowered 6-OHDA-induced apoptosis, as established by assessing levels of specific DNA denaturation by formamide, as well as the ratio of pro-apoptotic Bax and anti-apoptotic Bcl-2 expression, and reduced mitochondrial superoxide anion levels. 

The protective effects of hydroxytyrosol against toxins commonly used in PD research, including DA, 6-OHD, and MPP+ were evaluated on dopaminergic SH-SY5Y cells [179]. Hydroxytyrosol had a strong protective effect against DA- or 6-OHDA-induce cell death, but had little effect on MPP^+^-induced cytotoxicity suggesting that the cellular mechanisms underlying DA- or 6-OHDA-induced toxicity are different from that of MPP+. Moreover, 20 µM hydroxytyrosol induced phase II detoxifying enzymes such as NQO1, HO-1, glutathione *S*-transferase and the modifier subunit of glutamate cysteine ligase which catalyzes the first and rate-limiting step in the synthesis of GSH. Using an NQO1 inhibitor, the authors revealed that increased NQO1 expression contributed to the protective effect of hydroxytyrosol against dopaminergic cell death.

MAO-B inhibitors are used in the symptomatic treatment of Parkinson’s disease as they increase synaptic dopamine by blocking its degradation [170]. On the other hand, the rise in cytoplasmic dopamine leads to an increase of spontaneous oxidation to DA-quinone species, including 5-*S*-cysteinyl-dopamine (Cys-DA) [170,180], superoxide radicals and hydrogen peroxide [47]. With the purpose to mitigate the MAO inhibitor-induced increase in spontaneous DA oxidation, Goldstein et al. [181] investigated the effect of hydroxytyrosol on PC12 cells in the presence of different MAO inhibitors: clorgyline or the MAO-B inhibitors rasagiline or selegiline. The results clearly showed that hydroxytyrosol decreased Cys-DA levels induced by MAO inhibitors, suggesting that this olive oil phenol could be used to enhance the efficacy of the clinical treatment of Parkinson’s disease. Vauzour et al. [182] showed that tyrosol counteracted cell death induced by Cys-DA in primary cultures of mouse cortical neurons. Of note, they demonstrated that the protection evoked by tyrosol was equal to or greater than that observed for the flavonoids, (+)-catechin, (−)-epicatechin and quercetin.

Levodopa, the gold standard in the treatment of Parkinson’s disease, can be converted to DA in the periphery by catechol-*O*-methyl transferase (COMT), reducing the level of levodopa reaching the brain. For this reason, COMT inhibitors are used as adjuncts to levodopa therapy in Parkinson’s disease. In this context, Gallardo et al., investigated the inhibition of COMT activity by hydroxytyrosol, measuring intracellular dopamine and its metabolite levels in the corpus striatum of rats [156]. The animals received a single dose of 20 mg/kg or one daily dose of 20 mg/kg for 5 days of hydroxytyrosol. Both treatments produced a significant increase in the intracellular levels of DA and its metabolite, 3,4-dihydroxyphenylacetic acid, with the chronic treatment most effective that the acute one. 

Summarizing, in the context of Parkinson’s disease olive oil phenols are multitarget compounds able to modulate different cellular mechanism involved in the onset and progression of the disease. Most of the studies have been carried out in cell cultures, so further in vivo studies are absolutely needed to confirm the protective effects of olive oil phenols observed in vitro. Moreover, in our opinion, it would be important to explore the protective effect of olive oil as a whole in Parkinson’s disease. 

### 7.3. Multiple Sclerosis

MS is a chronic inflammatory and neurodegenerative disease of the central nervous system characterized by focal lesions of inflammation, BBB breakdown, axonal and oligodendrocyte injury, gliosis, and demyelination [183,184]. The incidence of MS has increased from 2.1 million in 2008 to 2.3 million in 2013 [185] and is now the first cause of disability after traumatic brain injury in young individuals [186]. In the pathogenesis of MS different parts of the immune system are involved including autoreactive T cells, macrophages and microglial cells, as well as antibodies and inflammation-related enzymes such as COX-2 and iNOS and cytokines such as IFN-γ, TNF-α or IL-1 [187]. Moreover, increasing evidence suggests that, due to the excessive generation of ROS, oxidative stress is one of the most important components involved in the pathogenesis of this disease [188,189]. Likewise other important neurodegenerative diseases, nowadays no definitive therapy are available for MS and conventional therapeutic approaches are related to many undesirable side effects [190]. 

It has been shown that the up-regulation of matrix metallopeptidase 9 (MMP-9) increases the permeability of BBB, facilitates the infiltration of leukocytes into the central nervous system, and leads to myelin degradation as well as neuronal damage in multiple sclerosis patients [191,192]. In this context, Liuzzi et al. [193] investigated the effect of an olive oil extract on the levels of MMP-9 and MMP-2 in rat astrocytes stimulated with LPS and in serum samples from multiple sclerosis patients. The results demonstrated that olive oil extract was able to reduce mRNA levels and activity of MMP-9 and MMP-2 enzymes both in activated astrocytes and serum of multiple sclerosis patients, suggesting that olive oil might be useful in inhibiting the activity of gelatinases involved in the course of the inflammatory responses observed in MS. 

To our knowledge no other studies have been carried out to investigate the effect of olive oil phenols in counteracting multiple sclerosis. Of note, two different studies explored the protective effects of two natural triterpenes present in olive oil, oleanolic acid and erythrodiol on experimental autoimmune encephalomyelitis (EAE) that is considered the best available model for understanding events in multiple sclerosis [194,195]. The prophylactic administration of triterpenes delayed the onset and decreased the severity of the disease ameliorating the neurological signs of EAE-mice, by preventing up-regulation of specific antibodies and inflammatory cytokines, and stabilizing the BBB integrity, thus reducing the migration of leukocytes in the central nervous system (CNS). 

Overall, olive oil consumption in the frame of the MD to ameliorate the wellness of MS patients is strongly suggested [196].

### 7.4. Amyotrophic Lateral Sclerosis

ALS is an adult-onset, progressive, and untreatable neurological disease characterized by selective loss of motor neurons in brain and spinal cord muscle weakness, which results in atrophy and spasticity, typically leading to paralysis and finally death because the respiratory muscles are compromised [197].

Although most cases of ALS are sporadic and of unknown etiology, less than 10% of patients have familial ALS, typically as an autosomal dominant trait [198]. The two forms of ALS are clinically indistinguishable, even if 15–20% of familial ALS patients carry mutations in the gene encoding for the antioxidant enzyme Cu/Zn SOD-1 [199,200]. The role of SOD-1 in ALS is not completely understood, but it has been suggested that the neurodegenerative disorder is due to gain of toxic function rather than loss of SOD1 enzymatic activity [201,202].

The mechanism of motor neuron degeneration is under intense investigation. There is increasing evidence that protein misfolding, mitochondrial dysfunction, oxidative radical damage, defective axonal transport, excitotoxicity, insufficient growth factor signaling and inflammation are responsible for the etiology of ALS [203,204]. ALS treatments is only palliative, and no drugs are available to halt the progression of the disease [205]. In this context, Oliván et al. [104] observed that olive oil ameliorated pathological outcomes and delayed the disease onset in an ALS model of mice overexpressing a mutant form of SOD-1 (SOD1G93A variant). Mice that received olive oil survived longer and showed better motor performance and larger muscle fiber area than animals receiving palm oil. In addition, olive oil supplementation improved the muscle status as demonstrated by the increased expression of myogenic factors (Myod1 and Myog) and autophagy markers (LC3 and Beclin1), as well as diminished endoplasmic reticulum stress through decreasing Atf6 and Grp78. Another study [206] investigated the effect of a phenolic extract obtained from a commercial Italian olive oil in modulating TLR4 signaling that has been reported to be involved in ALS pathogenic mechanisms [207]. The results proved that olive oil phenolic extract induced a complete inhibition of TLR4 activation, prevented the death of motoneurons induced by LPS and in motoneurons/glia co-cultures was very effective in protecting motoneurons from the toxicity of microglia carrying the SOD1G93A mutation. Olive oil phenols were also able to inhibit the release of nitric oxide induced by LPS treatment or SOD1mut glia, suggesting that olive oil phenols could have neuroprotective effects related to the modulation of inflammatory mediators.

## 8. Clinical Trials and Population Studies on Olive Oil and Its Phenols

Few clinical trials and population studies have been carried out to investigate the effects of olive oil and its phenols in counteracting neurodegeneration and most of them are related to the MD and its impact on cognitive decline. The “Three-City-Study” was the first to evidence an association between olive oil consumption and cognitive decline. The study was carried out on 8028 subjects aged 65 years and over recruited from three French cities. Participants with moderate or intensive use of olive oil compared to those who never used olive oil showed lower odds of cognitive deficit for verbal fluency and visual memory [208].

The PREDIMED-NAVARRA randomized trial assessed the effect on cognition of a nutritional intervention using a MD supplemented with extra-virgin olive oil in comparison with a low-fat control diet on 522 participants at high vascular risk. After 6.5 years of intervention the participants allocated to the MD plus extra virgin olive oil showed higher mean Mini-Mental State Examination and Clock Drawing Test scores with significant differences versus control, suggesting a protective effect of olive oil against age-related cognitive impairment [209]. A clinical trial aimed to evaluate the effect of vegetal oil on cognitive impairment in respect to olive oil as a placebo (ClinicalTrials.gov Identifier: NCT02778581 study start date March 2016) is now recruiting. The results will help to clarify if olive oil has a higher effect in improving cognition compared to other vegetable oils. 

In this context, more clinical studies are needed to understand the exact contribution of olive oil to the beneficial effect of the MD and, in particular, to elucidate the protective effect of olive oil and its phenols on neurological disorders. 

## 9. Conclusions

The beneficial effects of olive oil and its phenolics on neurological disorders have been extensively investigated and associated to the modulation of many different cell pathways. Among phenolics, oleocanthal, oleuropein, hydroxytyrosol and tyrosol have been mostly explored (Table 1 summarizes their effects against neurodegeneration). They possess a direct antioxidant activity in vitro, although their low concentration in vivo is not sufficient to justify a protective mechanism related to their ability to scavenge ROS. Evidence is arising suggesting that olive oil phenol effects in the brain could be mediated by the activation of the Keap1-Nrf2 pathway, a key regulator of the antioxidant system. Regarding acute brain injury, olive oil and its phenols have been shown to counteract stroke and spinal cord injury. In particular, olive oil, hydroxytyrosol, tyrosol and oleuropein demonstrated to reduce infarct volume, apoptosis and ameliorate the outcome of these injuries. Only oleuropein has been investigated in relation to spinal cord injury and its protective effect has been ascribed to its ability to reduce inflammation.

Concerning chronic neurodegeneration, the effect of olive oil and its phenolic compounds have been mainly investigated in relation to AD and PD. Olive oil phenols reduce both Aβ and neurofibrillary tangles deposition interfering at different levels with their production and clearance. Moreover, their ability to boost the antioxidant system and reduce inflammation has also been associated with their protective effect in AD, PD, MS, and ALS. 

To conclude, olive oil phenols, thanks to their multiple modes of action, have a great potential for therapeutic success in counteracting multifactorial pathologies such as neurological disorders. In this regard, further clinical trials are needed to provide a broader insight on the preventive/therapeutic potential and to identify biologically relevant concentrations.

## Figures and Tables

**Table 1:** Studies showing a protective activity of olive oil phenols against neurological disorders.

Olive Oil Phenols	Activity	References
Oleuropein	Counteraction of LDL oxidations	[85]
	Counteraction of oxidative stress at brain level	[88,91]
	Induction of autophagy at micromolar concentration	[100]
	Inhibition of autophagy at picomolar concentration	[103,104]
	Reduction of cerebral infarct volume after cerebral ischemia/reperfusion injury in mice	[115]
	Reduction of inflammatory biomarkers (TNF-α, IL-1α, iNOS, COX-2) after spinal cord injury (animal model)	[122,123,124]
	interference with amyloid aggregation (in vitro)	[32,138]
	Reduction of Aβ42 deposition and plaque deposit (animal models)	[3,139]
	Counteraction of pE3-Aβ production	[39,140]
	Reduction of oxidative stress and apoptosis induced by 6-OHDA (in vitro)	[103,178]
Oleocanthal	Inhibition of COX activity	[46]
	Anti-aggregation activities on tau protein (in vitro)	[147,148,149]
	Enhancement of Aβ clearance from the brain (animal model)	[38]
	Reduction of astrocytes activation and IL-1β levels in AD model	[31,150]
Hydroxytyrosol	Inhibition of COX activity	[84]
	Counteraction of oxidative stress at brain level	[86,91]
	Counteraction of hypoxic brain damage (ex-vivo model)	[111]
	Attenuates Aβ induced inflammation (animal model)	[145]
	Beneficial effects on insulin resistance associated with AD	[146]
	Protection against DA- or 6-OHDA-induce cell death (in vitro)	[179]
	Inhibition of COMT activity, leading to increased intracellular DA levels (animal model)	[156]
Tyrosol	Counteraction of hypoxic brain damage	[111]
	Attenuation of mitochondrial dysfunction and ATP depletion induced by MPP+ (in vitro)	[177]
	Counteraction of Cys-DA induced cytotoxicity (in vitro)	[182]

## Abbreviations

4-HNE	4-hydroxynoneal
6-OHDA	6-hydroxydopamine
ABCA1	ATP-binding cassette transporter 1
AD	Alzheimer’s disease
ALS	amyotrophic lateral sclerosis
APOE	apolipoprotein E
APP	amyloid precursor protein
Aβ	amyloid-β
BBB	blood brain barrier
CAT	catalase
CBVD	cerebrovascular disease
CNS	central nervous system
COMT	catechol-*O*-methyl transferase
COX	cyclooxygenases
Cys-DA	5-*S*-cysteinyl-dopamine
DA	dopamine
EAE	autoimmune encephalomyelitis
EEG	electroencephalography
GFAP	glial fibrillary acidic protein
GLT1	glutamine transporter 1
GLUT1	glucose transporter 1
GPx	glutathione peroxidase
GR	glutathione reductase
HD	Huntington’s disease
HO-1	heme oxygenase 1
IL-1β	interleukin-1β
iNOS	inducible nitric oxide synthase
Keap1	Kelch-like-ECH-associated protein 1
LC3	protein 1 light chain 3
l-DOPA	l-3,4-dihydroxyphenylalanine
LRP1	LDL receptor related protein-1
MAO	monoamine oxidase
MCAO	middle cerebral artery occlusion
MD	Mediterranean diet
MDA	Malondialdehyde
MMP-9	matrix metallopeptidase 9
MPO	myeloperoxidase
MPP+	methyl-4-phenylpyridinium
MS	multiple sclerosis
mTOR	mammalian target of rapamycin
NF	nuclear factor
NFTs	neurofibrillary tangles
NGF	nerve growth factor
NGF	nerve growth factor
NO	nitric oxide
NQO1	NADPH quinone oxidoreductase 1
Nrf2	nuclear factor NF-E2-related factor 2
PD	Parkinson’s disease
pE3-Aβ	pyroglutamate-modified Aβ peptides at amino acid position three
PGE2	prostaglandin E2
P-gp	*P*-glycoprotein
PINK1	PTEN-induced putative kinase 1
PPARγ	peroxisome proliferator–activated receptor gamma
PS1	presenilin-1
PS2	presenilin-2
PSD-95	postsynaptic density protein 95
PUFA	polyunsaturated fatty acid
ROS	reactive oxygen species
SCI	spinal cord injury
SOD	superoxide dismutase
TBARS	thiobarbituric Acid Reactive Substances
TNF	tumor necrosis factor
ULK1	unc-51-like kinase 1

## References

[1] Ritchie K., Lovestone S. (2002). The dementias. Lancet.

[2] Akhlaq A., Farooqui A. (2010). Neurochemical Aspects of Neurotraumatic and Neurodegenerative Diseases.

[3] Liu Z., Zhou T., Ziegler A.C., Dimitrion P., Zuo L. (2017). Oxidative Stress in Neurodegenerative Diseases: From Molecular Mechanisms to Clinical Applications. Oxid. Med. Cell. Longev..

[4] Ferri C.P., Prince M., Brayne C., Brodaty H., Fratiglioni L., Ganguli M., Hall K., Hasegawa K., Hendrie H., Huang Y. (2005). Global prevalence of dementia: A Delphi consensus study. Lancet.

[5] Kukull W.A., Higdon R., Bowen J.D. (2002). Dementia and Alzheimer disease incidence: A prospective cohort study. Arch. Neurol..

[6] De Rijk M.C., Launer L.J., Berger K., Breteler M.M., Dartigues J.F., Baldereschi M., Fratiglioni L., Lobo A., Martinez-Lage J., Trenkwalder C. (2000). Prevalence of Parkinson’s disease in Europe: A collaborative study of population-based cohorts. Neurologic Diseases in the Elderly Research Group. Neurology.

[7] Farrer M.J. (2006). Genetics of Parkinson disease: Paradigm shifts and future prospects. Nat. Rev. Genet..

[8] Rouanet C., Reges D., Rocha E., Gagliardi V., Silva G.S. (2017). Traumatic spinal cord injury: Current concepts and treatment update. Arq. Neuropsiquiatr..

[9] Cruz-Haces M., Tang J., Acosta G., Fernandez J., Shi R. (2017). Pathological correlations between traumatic brain injury and chronic neurodegenerative diseases. Transl. Neurodegener..

[10] Lee P.C., Bordelon Y., Bronstein J., Ritz B. (2012). Traumatic brain injury, paraquat exposure, and their relationship to Parkinson disease. Neurology.

[11] Campdelacreu J. (2012). Parkinson disease and Alzheimer disease: Environmental risk factors. Neurologia.

[12] Pluta R. (2004). From brain ischemia-reperfusion injury to possible sporadic Alzheimer’s disease. Curr. Neurovasc. Res..

[13] Mandel S., Grunblatt E., Riederer P., Gerlach M., Levites Y., Youdim M.B. (2003). Neuroprotective strategies in Parkinson’s disease: An update on progress. CNS Drugs.

[14] Dauer W., Przedborski S. (2003). Parkinson’s disease: Mechanisms and models. Neuron.

[15] Tarozzi A., Angeloni C., Malaguti M., Morroni F., Hrelia S., Hrelia P. (2013). Sulforaphane as a potential protective phytochemical against neurodegenerative diseases. Oxid. Med. Cell. Longev..

[16] Vauzour D., Buonfiglio M., Corona G., Chirafisi J., Vafeiadou K., Angeloni C., Hrelia S., Hrelia P., Spencer J.P. (2010). Sulforaphane protects cortical neurons against 5-S-cysteinyl-dopamine-induced toxicity through the activation of ERK1/2, Nrf-2 and the upregulation of detoxification enzymes. Mol. Nutr. Food Res..

[17] Vauzour D., Ravaioli G., Vafeiadou K., Rodriguez-Mateos A., Angeloni C., Spencer J.P. (2008). Peroxynitrite induced formation of the neurotoxins 5-S-cysteinyl-dopamine and DHBT-1: Implications for Parkinson’s disease and protection by polyphenols. Arch. Biochem. Biophys..

[18] Aruoma O.I., Bahorun T., Jen L.S. (2003). Neuroprotection by bioactive components in medicinal and food plant extracts. Mutat. Res..

[19] Vauzour D., Vafeiadou K., Rodriguez-Mateos A., Rendeiro C., Spencer J.P. (2008). The neuroprotective potential of flavonoids: A multiplicity of effects. Genes Nutr..

[20] Kelsey N.A., Wilkins H.M., Linseman D.A. (2010). Nutraceutical antioxidants as novel neuroprotective agents. Molecules.

[21] Féart C., Samieri C., Allès B., Barberger-Gateau P. (2013). Potential benefits of adherence to the Mediterranean diet on cognitive health. Proc. Nutr. Soc..

[22] Scarmeas N., Stern Y., Tang M.X., Mayeux R., Luchsinger J.A. (2006). Mediterranean diet and risk for Alzheimer’s disease. Ann. Neurol..

[23] Scarmeas N., Luchsinger J.A., Stern Y., Gu Y., He J., De Carli C., Brown T., Brickman A.M. (2011). Mediterranean diet and magnetic resonance imaging-assessed cerebrovascular disease. Ann. Neurol..

[24] Féart C., Samieri C., Barberger-Gateau P. (2010). Mediterranean diet and cognitive function in older adults. Curr. Opin. Clin. Nutr. Metab. Care.

[25] Scarmeas N., Stern Y., Mayeux R., Manly J.J., Schupf N., Luchsinger J.A. (2009). Mediterranean diet and mild cognitive impairment. Arch. Neurol..

[26] Middleton E. (1998). Effect of plant flavonoids on immune and inflammatory cell function. Adv. Exp. Med. Biol..

[27] Hollman P.C., Katan M.B. (1999). Health effects and bioavailability of dietary flavonols. Free Radic. Res..

[28] Eastwood M.A. (1999). Interaction of dietary antioxidants in vivo: How fruit and vegetables prevent disease?. QJM Mon. J. Assoc. Phys..

[29] Pitozzi V., Jacomelli M., Catelan D., Servili M., Taticchi A., Biggeri A., Dolara P., Giovannelli L. (2012). Long-term dietary extra-virgin olive oil rich in polyphenols reverses age-related dysfunctions in motor coordination and contextual memory in mice: Role of oxidative stress. Rejuvenation Res..

[30] López-Miranda J., Pérez-Jiménez F., Ros E., De Caterina R., Badimón L., Covas M.I., Escrich E., Ordovás J.M., Soriguer F., Abiá R. (2010). Olive oil and health: Summary of the II international conference on olive oil and health consensus report, Jaén and Córdoba (Spain) 2008. Nutr. Metab. Cardiovasc. Dis..

[31] Qosa H., Mohamed L.A., Batarseh Y.S., Alqahtani S., Ibrahim B., LeVine H., Keller J.N., Kaddoumi A. (2015). Extra-virgin olive oil attenuates amyloid-β and tau pathologies in the brains of TgSwDI mice. J. Nutr. Biochem..

[32] Rigacci S., Guidotti V., Bucciantini M., Nichino D., Relini A., Berti A., Stefani M. (2011). Aβ(1–42) aggregates into non-toxic amyloid assemblies in the presence of the natural polyphenol oleuropein aglycon. Curr. Alzheimer Res..

[33] Pitt J., Roth W., Lacor P., Smith A.B., Blankenship M., Velasco P., De Felice F., Breslin P., Klein W.L. (2009). Alzheimer’s-associated Aβ oligomers show altered structure, immunoreactivity and synaptotoxicity with low doses of oleocanthal. Toxicol. Appl. Pharmacol..

[34] Casamenti F., Stefani M. (2017). Olive polyphenols: New promising agents to combat aging-associated neurodegeneration. Expert Rev. Neurother..

[35] Singh M., Arseneault M., Sanderson T., Murthy V., Ramassamy C. (2008). Challenges for research on polyphenols from foods in Alzheimer’s disease: Bioavailability, metabolism, and cellular and molecular mechanisms. J. Agric. Food Chem..

[36] Kim H.S., Quon M.J., Kim J.A. (2014). New insights into the mechanisms of polyphenols beyond antioxidant properties; lessons from the green tea polyphenol, epigallocatechin 3-gallate. Redox Biol..

[37] Goszcz K., Duthie G.G., Stewart D., Leslie S.J., Megson I.L. (2017). Bioactive polyphenols and cardiovascular disease: Chemical antagonists, pharmacological agents or xenobiotics that drive an adaptive response?. Br. J. Pharmacol..

[38] Abuznait A.H., Qosa H., Busnena B.A., El Sayed K.A., Kaddoumi A. (2013). Olive-oil-derived oleocanthal enhances β-amyloid clearance as a potential neuroprotective mechanism against Alzheimer’s disease: In vitro and in vivo studies. ACS Chem. Neurosci..

[39] Grossi C., Rigacci S., Ambrosini S., Dami T.E., Luccarini I., Traini C., Failli P., Berti A., Casamenti F., Stefani M. (2013). The polyphenol oleuropein aglycone protects TgCRND8 mice against Aβ plaque pathology. PLoS ONE.

[40] Declerck K., Szarc vel Szic K., Palagani A., Heyninck K., Haegeman G., Morand C., Milenkovic D., Vanden Berghe W. (2016). Epigenetic control of cardiovascular health by nutritional polyphenols involves multiple chromatin-modifying writer-reader-eraser proteins. Curr. Top. Med. Chem..

[41] Ayissi V.B., Ebrahimi A., Schluesenner H. (2014). Epigenetic effects of natural polyphenols: A focus on SIRT1-mediated mechanisms. Mol. Nutr. Food Res..

[42] Servili M., Montedoro G. (2002). Contribution of phenolic compounds to virgin olive oil quality. Eur. J. Lipid Sci. Technol..

[43] Bendini A., Cerretani L., Alegria C.-P., Ana Maria G.-C., Antonio S.-C., Alberto F.-G., Lercker G. (2007). Phenolic molecules in virgin olive oils: A survey of their sensory properties, health effects, antioxidant activity and analytical methods. An overview of the last decade. Molecules.

[44] Servili M., Esposto S., Fabiani R., Urbani S., Taticchi A., Mariucci F., Selvaggini R., Montedoro G.F. (2009). Phenolic compounds in olive oil: Antioxidant, health and organoleptic activities according to their chemical structure. Inflammopharmacology.

[45] Rodríguez-Morató J., Xicota L., Fitó M., Farré M., Dierssen M., De la Torre R. (2015). Potential role of olive oil phenolic compounds in the prevention of neurodegenerative diseases. Molecules.

[46] Beauchamp G.K., Keast R.S., Morel D., Lin J., Pika J., Han Q., Lee C.H., Smith A.B., Breslin P.A. (2005). Phytochemistry: Ibuprofen-like activity in extra-virgin olive oil. Nature.

[47] Jenner P. (2003). Oxidative stress in Parkinson’s disease. Ann. Neurol..

[48] Kalogeropoulos N., Tsimidou M.Z. (2014). Antioxidants in Greek Virgin Olive Oils. Antioxidants (Basel).

[49] Lee S.Y., Moon Y., Hee Choi D., Jin Choi H., Hwang O. (2007). Particular vulnerability of rat mesencephalic dopaminergic neurons to tetrahydrobiopterin: Relevance to Parkinson’s disease. Neurobiol. Dis..

[50] De la Torre R. (2008). Bioavailability of olive oil phenolic compounds in humans. Inflammopharmacology.

[51] Vissers M.N., Zock P.L., Roodenburg A.J., Leenen R., Katan M.B. (2002). Olive oil phenols are absorbed in humans. J. Nutr..

[52] Visioli F., Galli C., Bornet F., Mattei A., Patelli R., Galli G., Caruso D. (2000). Olive oil phenolics are dose-dependently absorbed in humans. FEBS Lett..

[53] Miro-Casas E., Covas M.I., Farre M., Fito M., Ortuño J., Weinbrenner T., Roset P., De La Torre R. (2003). Hydroxytyrosol disposition in humans. Clin. Chem..

[54] Weinbrenner T., Fitó M., Farré Albaladejo M., Saez G.T., Rijken P., Tormos C., Coolen S., De La Torre R., Covas M.I. (2004). Bioavailability of phenolic compounds from olive oil and oxidative/antioxidant status at postprandial state in healthy humans. Drugs Exp. Clin. Res..

[55] Miró Casas E., Farré Albadalejo M., Covas Planells M.I., Fitó Colomer M., Lamuela Raventós R.M., De la Torre Fornell R. (2001). Tyrosol bioavailability in humans after ingestion of virgin olive oil. Clin. Chem..

[56] Miró-Casas E., Farré Albaladejo M., Covas M.I., Rodriguez J.O., Menoyo Colomer E., Lamuela Raventós R.M., De La Torre R. (2001). Capillary gas chromatography-mass spectrometry quantitative determination of hydroxytyrosol and tyrosol in human urine after olive oil intake. Anal. Biochem..

[57] González-Santiago M., Fonollá J., Lopez-Huertas E. (2010). Human absorption of a supplement containing purified hydroxytyrosol, a natural antioxidant from olive oil, and evidence for its transient association with low-density lipoproteins. Pharmacol. Res..

[58] D’Angelo S., Manna C., Migliardi V., Mazzoni O., Morrica P., Capasso G., Pontoni G., Galletti P., Zappia V. (2001). Pharmacokinetics and metabolism of hydroxytyrosol, a natural antioxidant from olive oil. Drug Metab. Dispos..

[59] Tuck K.L., Hayball P.J., Stupans I. (2002). Structural characterization of the metabolites of hydroxytyrosol, the principal phenolic component in olive oil, in rats. J. Agric. Food Chem..

[60] Lee M.J., Maliakal P., Chen L., Meng X., Bondoc F.Y., Prabhu S., Lambert G., Mohr S., Yang C.S. (2002). Pharmacokinetics of tea catechins after ingestion of green tea and (−)-epigallocatechin-3-gallate by humans: Formation of different metabolites and individual variability. Cancer Epidemiol. Biomark. Prev..

[61] Serra A., Rubió L., Borràs X., Macià A., Romero M.P., Motilva M.J. (2012). Distribution of olive oil phenolic compounds in rat tissues after administration of a phenolic extract from olive cake. Mol. Nutr. Food Res..

[62] García-Villalba R., Carrasco-Pancorbo A., Nevedomskaya E., Mayboroda O.A., Deelder A.M., Segura-Carretero A., Fernández-Gutiérrez A. (2010). Exploratory analysis of human urine by LC-ESI-TOF MS after high intake of olive oil: Understanding the metabolism of polyphenols. Anal. Bioanal. Chem..

[63] James D., Devaraj S., Bellur P., Lakkanna S., Vicini J., Boddupalli S. (2012). Novel concepts of broccoli sulforaphanes and disease: Induction of phase II antioxidant and detoxification enzymes by enhanced-glucoraphanin broccoli. Nutr. Rev..

[64] Navarro A., Boveris A. (2009). Brain mitochondrial dysfunction and oxidative damage in Parkinson’s disease. J. Bioenerg. Biomembr..

[65] Navarro S.L., Li F., Lampe J.W. (2011). Mechanisms of action of isothiocyanates in cancer chemoprevention: An update. Food Funct..

[66] Fahey J.W., Zalcmann A.T., Talalay P. (2001). The chemical diversity and distribution of glucosinolates and isothiocyanates among plants. Phytochemistry.

[67] Manoharan S., Guillemin G.J., Abiramasundari R.S., Essa M.M., Akbar M., Akbar M.D. (2016). The Role of Reactive Oxygen Species in the Pathogenesis of Alzheimer’s Disease, Parkinson’s Disease, and Huntington’s Disease: A Mini Review. Oxid. Med. Cell. Longev..

[68] Shapiro T.A., Fahey J.W., Wade K.L., Stephenson K.K., Talalay P. (1998). Human metabolism and excretion of cancer chemoprotective glucosinolates and isothiocyanates of cruciferous vegetables. Cancer Epidemiol. Biomark. Prev..

[69] Prakash C., Soni M., Kumar V. (2016). Mitochondrial oxidative stress and dysfunction in arsenic neurotoxicity: A review. J. Appl.Toxicol..

[70] Bones A.M., Rossiter J.T. (2006). The enzymic and chemically induced decomposition of glucosinolates. Phytochemistry.

[71] Halkier B.A., Gershenzon J. (2006). Biology and biochemistry of glucosinolates. Annu. Rev. Plant Biol..

[72] Subramaniam S.R., Federoff H.J. (2017). Targeting Microglial Activation States as a Therapeutic Avenue in Parkinson’s Disease. Front. Aging Neurosci..

[73] Ransohoff R.M. (2016). How neuroinflammation contributes to neurodegeneration. Science.

[74] Cooper D.A., Webb D.R., Peters J.C. (1997). Evaluation of the potential for olestra to affect the availability of dietary phytochemicals. J. Nutr..

[75] Matusheski N.V., Juvik J.A., Jeffery E.H. (2004). Heating decreases epithiospecifier protein activity and increases sulforaphane formation in broccoli. Phytochemistry.

[76] Verkerk R., Schreiner M., Krumbein A., Ciska E., Holst B., Rowland I., De Schrijver R., Hansen M., Gerhäuser C., Mithen R. (2009). Glucosinolates in Brassica vegetables: The influence of the food supply chain on intake, bioavailability and human health. Mol. Nutr. Food Res..

[77] Jakubikova J., Sedlak J., Mithen R., Bao Y. (2005). Role of PI3K/Akt and MEK/ERK signaling pathways in sulforaphane- and erucin-induced phase II enzymes and MRP2 transcription, G2/M arrest and cell death in Caco-2 cells. Biochem. Pharmacol..

[78] Hanlon N., Coldham N., Sauer M.J., Ioannides C. (2009). Modulation of rat pulmonary carcinogen-metabolising enzyme systems by the isothiocyanates erucin and sulforaphane. Chem. Biol. Interact..

[79] Melchini A., Costa C., Traka M., Miceli N., Mithen R., De Pasquale R., Trovato A. (2009). Erucin, a new promising cancer chemopreventive agent from rocket salads, shows anti-proliferative activity on human lung carcinoma A549 cells. Food Chem. Toxicol..

[80] Kassahun K., Davis M., Hu P., Martin B., Baillie T. (1997). Biotransformation of the naturally occurring isothiocyanate sulforaphane in the rat: Identification of phase I metabolites and glutathione conjugates. Chem. Res. Toxicol..

[81] Ganguly G., Chakrabarti S., Chatterjee U., Saso L. (2017). Proteinopathy, oxidative stress and mitochondrial dysfunction: Cross talk in Alzheimer’s disease and Parkinson’s disease. Drug Des. Dev. Ther..

[82] Visioli F., Poli A., Gall C. (2002). Antioxidant and other biological activities of phenols from olives and olive oil. Med. Res. Rev..

[83] Petroni A., Blasevich M., Salami M., Papini N., Montedoro G.F., Galli C. (1995). Inhibition of platelet aggregation and eicosanoid production by phenolic components of olive oil. Thromb. Res..

[84] González-Correa J.A., Navas M.D., Muñoz-Marín J., Trujillo M., Fernández-Bolaños J., De La Cruz J.P. (2008). Effects of hydroxytyrosol and hydroxytyrosol acetate administration to rats on platelet function compared to acetylsalicylic acid. J. Agric. Food Chem..

[85] Visioli F., Galli C. (1994). Oleuropein protects low density lipoprotein from oxidation. Life Sci..

[86] Schaffer S., Podstawa M., Visioli F., Bogani P., Müller W.E., Eckert G.P. (2007). Hydroxytyrosol-rich olive mill wastewater extract protects brain cells in vitro and ex vivo. J. Agric. Food Chem..

[87] Majhi C.R., Khan S., Leo M.D., Manimaran A., Sankar P., Sarkar S.N. (2011). Effects of acetaminophen on reactive oxygen species and nitric oxide redox signaling in kidney of arsenic-exposed rats. Food Chem. Toxicol..

[88] Ogun M., Ozcan A., Karaman M., Karapehlivan M. (2016). Oleuropein ameliorates arsenic induced oxidative stress in mice. J. Trace Elem. Med. Biol..

[89] Soni M., Prakash C., Sehwag S., Kumar V. (2017). Protective effect of hydroxytyrosol in arsenic-induced mitochondrial dysfunction in rat brain. J. Biochem. Mol. Toxicol..

[90] González-Correa J.A., Muñoz-Marín J., Arrebola M.M., Guerrero A., Narbona F., López-Villodres J.A., De La Cruz J.P. (2007). Dietary virgin olive oil reduces oxidative stress and cellular damage in rat brain slices subjected to hypoxia-reoxygenation. Lipids.

[91] Peng S., Zhang B., Yao J., Duan D., Fang J. (2015). Dual protection of hydroxytyrosol, an olive oil polyphenol, against oxidative damage in PC12 cells. Food Funct..

[92] Sun W., Wang X., Hou C., Yang L., Li H., Guo J., Huo C., Wang M., Miao Y., Liu J., Kang Y. (2017). Oleuropein improves mitochondrial function to attenuate oxidative stress by activating the Nrf2 pathway in the hypothalamic paraventricular nucleus of spontaneously hypertensive rats. Neuropharmacology.

[93] Schaffer S., Halliwell B. (2012). Do polyphenols enter the brain and does it matter? Some theoretical and practical considerations. Genes Nutr..

[94] Yasuhara T., Hara K., Sethi K.D., Morgan J.C., Borlongan C.V. (2007). Increased 8-OHdG levels in the urine, serum, and substantia nigra of hemiparkinsonian rats. Brain Res..

[95] Ciechanover A. (2005). Proteolysis: From the lysosome to ubiquitin and the proteasome. Nat. Rev. Mol. Cell Biol..

[96] Reggiori F., Komatsu M., Finley K., Simonsen A. (2012). Autophagy: More than a nonselective pathway. Int. J. Cell. Biol..

[97] Vidal R.L., Matus S., Bargsted L., Hetz C. (2014). Targeting autophagy in neurodegenerative diseases. Trends Pharmacol. Sci..

[98] Choi A.M., Ryter S.W., Levine B. (2013). Autophagy in human health and disease. N. Engl. J. Med..

[99] Lamb C.A., Yoshimori T., Tooze S.A. (2013). The autophagosome: Origins unknown, biogenesis complex. Nat. Rev. Mol. Cell Biol..

[100] Rigacci S., Miceli C., Nediani C., Berti A., Cascella R., Pantano D., Nardiello P., Luccarini I., Casamenti F., Stefani M. (2015). Oleuropein aglycone induces autophagy via the AMPK/mTOR signalling pathway: A mechanistic insight. Oncotarget.

[101] Bhutani N., Piccirillo R., Hourez R., Venkatraman P., Goldberg A.L. (2012). Cathepsins L and Z are critical in degrading polyglutamine-containing proteins within lysosomes. J. Biol. Chem..

[102] Rubinsztein D.C., Codogno P., Levine B. (2012). Autophagy modulation as a potential therapeutic target for diverse diseases. Nat. Rev. Drug Discov..

[103] Achour I., Arel-Dubeau A.M., Renaud J., Legrand M., Attard E., Germain M., Martinoli M.G. (2016). Oleuropein Prevents Neuronal Death, Mitigates Mitochondrial Superoxide Production and Modulates Autophagy in a Dopaminergic Cellular Model. Int. J. Mol. Sci..

[104] Oliván S., Martínez-Beamonte R., Calvo A.C., Surra J.C., Manzano R., Arnal C., Osta R., Osada J. (2014). Extra virgin olive oil intake delays the development of amyotrophic lateral sclerosis associated with reduced reticulum stress and autophagy in muscle of SOD1G93A mice. J. Nutr. Biochem..

[105] Coux O., Tanaka K., Goldberg A.L. (1996). Structure and functions of the 20S and 26S proteasomes. Annu. Rev. Biochem..

[106] Hochstrasser M. (1992). Ubiquitin and intracellular protein degradation. Curr. Opin. Cell Biol..

[107] McKinnon C., Tabrizi S.J. (2014). The ubiquitin-proteasome system in neurodegeneration. Antioxid. Redox Signal..

[108] Katsiki M., Chondrogianni N., Chinou I., Rivett A.J., Gonos E.S. (2007). The olive constituent oleuropein exhibits proteasome stimulatory properties in vitro and confers life span extension of human embryonic fibroblasts. Rejuvenation Res..

[109] Lipton P. (1999). Ischemic cell death in brain neurons. Physiol. Rev..

[110] Ham P.B., Raju R. (2016). Mitochondrial function in hypoxic ischemic injury and influence of aging. Prog. Neurobiol..

[111] González-Correa J.A., Navas M.D., Lopez-Villodres J.A., Trujillo M., Espartero J.L., De La Cruz J.P. (2008). Neuroprotective effect of hydroxytyrosol and hydroxytyrosol acetate in rat brain slices subjected to hypoxia-reoxygenation. Neurosci. Lett..

[112] Mohagheghi F., Bigdeli M.R., Rasoulian B., Zeinanloo A.A., Khoshbaten A. (2010). Dietary virgin olive oil reduces blood brain barrier permeability, brain edema, and brain injury in rats subjected to ischemia-reperfusion. Sci. World J..

[113] Rabiei Z., Bigdeli M.R., Rasoulian B. (2013). Neuroprotection of dietary virgin olive oil on brain lipidomics during stroke. Curr. Neurovasc. Res..

[114] Bu Y., Rho S., Kim J., Kim M.Y., Lee D.H., Kim S.Y., Choi H., Kim H. (2007). Neuroprotective effect of tyrosol on transient focal cerebral ischemia in rats. Neurosci. Lett..

[115] Yu H., Liu P., Tang H., Jing J., Lv X., Chen L., Jiang L., Xu J., Li J. (2016). Oleuropein, a natural extract from plants, offers neuroprotection in focal cerebral ischemia/reperfusion injury in mice. Eur. J. Pharmacol..

[116] Misirli G., Benetou V., Lagiou P., Bamia C., Trichopoulos D., Trichopoulou A. (2012). Relation of the traditional Mediterranean diet to cerebrovascular disease in a Mediterranean population. Am. J. Epidemiol..

[117] Ahuja C.S., Wilson J.R., Nori S., Kotter M.R.N., Druschel C., Curt A., Fehlings M.G. (2017). Traumatic spinal cord injury. Nat. Rev. Dis Primers.

[118] Profyris C., Cheema S.S., Zang D., Azari M.F., Boyle K., Petratos S. (2004). Degenerative and regenerative mechanisms governing spinal cord injury. Neurobiol. Dis..

[119] Amar A.P., Levy M.L. (1999). Pathogenesis and pharmacological strategies for mitigating secondary damage in acute spinal cord injury. Neurosurgery.

[120] Fleming J.C., Norenberg M.D., Ramsay D.A., Dekaban G.A., Marcillo A.E., Saenz A.D., Pasquale-Styles M., Dietrich W.D., Weaver L.C. (2006). The cellular inflammatory response in human spinal cords after injury. Brain.

[121] Taoka Y., Okajima K., Uchiba M., Murakami K., Kushimoto S., Johno M., Naruo M., Okabe H., Takatsuki K. (1997). Role of neutrophils in spinal cord injury in the rat. Neuroscience.

[122] Khalatbary A.R., Ahmadvand H. (2011). Effect of oleuropein on tissue myeloperoxidase activity in experimental spinal cord trauma. Iran. Biomed. J..

[123] Impellizzeri D., Esposito E., Mazzon E., Paterniti I., Di Paola R., Bramanti P., Morittu V.M., Procopio A., Perri E., Britti D. (2012). The effects of a polyphenol present in olive oil, oleuropein aglycone, in an experimental model of spinal cord injury in mice. Biochem. Pharmacol..

[124] Khalatbary A.R., Ahmadvand H. (2012). Neuroprotective effect of oleuropein following spinal cord injury in rats. Neurol. Res..

[125] Citron M. (2010). Alzheimer’s disease: Strategies for disease modification. Nat. Rev. Drug Discov..

[126] Walsh D.M., Selkoe D.J. (2004). Deciphering the molecular basis of memory failure in Alzheimer’s disease. Neuron.

[127] Selkoe D.J. (2002). Alzheimer’s disease is a synaptic failure. Science.

[128] Querfurth H.W., LaFerla F.M. (2010). Alzheimer’s disease. N. Engl. J. Med..

[129] Hort J., O’Brien J.T., Gainotti G., Gurvit H., Nacmias B., Pasquier F., Popescu O., Rektorova I., Religa D., Tusina R. (2010). EFNS guidelines for the diagnosis and management of Alzheimer’s disease. Eur. J. Neurol..

[130] Kozlov S., Afonin A., Evsyukov I., Bondarenko A. (2017). Alzheimer’s disease: As it was in the beginning. Rev. Neurosci..

[131] Markesbery W.R., Carney J.M. (1999). Oxidative alterations in Alzheimer’s disease. Brain Pathol..

[132] Angeloni C., Zambonin L., Hrelia S. (2014). Role of methylglyoxal in Alzheimer’s disease. BioMed Res. Int..

[133] Saleem M., Herrmann N., Swardfager W., Eisen R., Lanctôt K.L. (2015). Inflammatory Markers in Mild Cognitive Impairment: A Meta-Analysis. J. Alzheimers Dis..

[134] Scarmeas N., Stern Y., Mayeux R., Luchsinger J.A. (2006). Mediterranean diet, Alzheimer disease, and vascular mediation. Arch. Neurol..

[135] Pitozzi V., Jacomelli M., Zaid M., Luceri C. (2010). Effects of dietary extra-virgin olive oil on behaviour and brain biochemical parameters in ageing rats. Br. J. Nutr..

[136] Valls-Pedret C., Lamuela-Raventós R.M., Medina-Remón A., Martinez-Gonzalez M.A., Medina-Remon A., Pinto X., Quintana M., Ros E., Valls-Pedret C. (2012). Polyphenol-rich foods in the Mediterranean diet are associated with better cognitive function in elderly subjects at high cardiovascular risk. J. Alzheimers Dis..

[137] Farr S.A., Price T.O., Dominguez L.J., Motisi A., Saiano F., Niehoff M.L., Morley J.E., Banks W.A., Ercal N., Barbagallo M. (2012). Extra virgin olive oil improves learning and memory in SAMP8 mice. J. Alzheimers Dis..

[138] Rigacci S., Guidotti V., Bucciantini M., Parri M., Nediani C., Cerbai E., Stefani M., Berti A. (2010). Oleuropein aglycon prevents cytotoxic amyloid aggregation of human amylin. J. Nutr. Biochem..

[139] Diomede L., Rigacci S., Romeo M., Stefani M., Salmona M. (2013). Oleuropein aglycone protects transgenic *C. elegans* strains expressing Aβ42 by reducing plaque load and motor deficit. PLoS ONE.

[140] Luccarini I., Grossi C., Rigacci S., Coppi E., Pugliese A.M., Pantano D., la Marca G., Ed Dami T., Berti A., Stefani M. (2015). Oleuropein aglycone protects against pyroglutamylated-3 amyloid-ß toxicity: Biochemical, epigenetic and functional correlates. Neurobiol. Aging.

[141] Pantano D., Luccarini I., Nardiello P., Servili M., Stefani M., Casamenti F. (2017). Oleuropein aglycone and polyphenols from olive mill waste water ameliorate cognitive deficits and neuropathology. Br. J. Clin. Pharmacol..

[142] Luccarini I., Ed Dami T., Grossi C., Rigacci S., Stefani M., Casamenti F. (2014). Oleuropein aglycone counteracts Aβ42 toxicity in the rat brain. Neurosci. Lett..

[143] Pourkhodadad S., Alirezaei M., Moghaddasi M., Ahmadvand H., Karami M., Delfan B., Khanipour Z. (2016). Neuroprotective effects of oleuropein against cognitive dysfunction induced by colchicine in hippocampal CA1 area in rats. J. Physiol. Sci..

[144] St-Laurent-Thibault C., Arseneault M., Longpré F., Ramassamy C. (2011). Tyrosol and hydroxytyrosol, two main components of olive oil, protect N2a cells against amyloid-β-induced toxicity. Involvement of the NF-κB signaling. Curr. Alzheimer Res..

[145] Peng Y., Hou C., Yang Z., Li C., Jia L., Liu J., Tang Y., Shi L., Li Y., Long J. (2016). Hydroxytyrosol mildly improve cognitive function independent of APP processing in APP/PS1 mice. Mol. Nutr. Food Res..

[146] Crespo M.C., Tomé-Carneiro J., Pintado C., Dávalos A., Visioli F., Burgos-Ramos E. (2017). Hydroxytyrosol restores proper insulin signaling in an astrocytic model of Alzheimer’s disease. Biofactors.

[147] Li W., Sperry J.B., Crowe A., Trojanowski J.Q., Smith A.B., Lee V.M. (2009). Inhibition of tau fibrillization by oleocanthal via reaction with the amino groups of tau. J. Neurochem..

[148] Monti M.C., Margarucci L., Tosco A., Riccio R., Casapullo A. (2011). New insights on the interaction mechanism between tau protein and oleocanthal, an extra-virgin olive-oil bioactive component. Food Funct..

[149] Monti M.C., Margarucci L., Riccio R., Casapullo A. (2012). Modulation of tau protein fibrillization by oleocanthal. J. Nat. Prod..

[150] Batarseh Y.S., Mohamed L.A., Al Rihani S.B., Kaddoumi A. (2017). Oleocanthal ameliorates amyloid-β oligomers’ toxicity on astrocytes and neuronal cells: In vitro studies. Neuroscience.

[151] Safouris A., Tsivgoulis G., Sergentanis T.N., Psaltopoulou T. (2015). Mediterranean Diet and Risk of Dementia. Curr. Alzheimer Res..

[152] Bellucci A., Mercuri N.B., Venneri A., Faustini G., Longhena F., Pizzi M., Missale C., Spano P. (2016). Review: Parkinson’s disease: From synaptic loss to connectome dysfunction. Neuropathol. Appl. Neurobiol..

[153] Rodriguez-Oroz M.C., Jahanshahi M., Krack P., Litvan I., Macias R., Bezard E., Obeso J.A. (2009). Initial clinical manifestations of Parkinson’s disease: Features and pathophysiological mechanisms. Lancet Neurol..

[154] Lotharius J., Brundin P. (2002). Pathogenesis of Parkinson’s disease: Dopamine, vesicles and α-synuclein. Nat. Rev. Neurosci..

[155] Sherer T.B., Betarbet R., Greenamyre J.T. (2001). Pathogenesis of Parkinson’s disease. Curr. Opin. Investig. Drugs.

[156] Gallardo E., Madrona A., Palma-Valdes R., Trujillo M., Espartero J.L., Santiago M. (2014). The effect of hydroxytyrosol and its nitroderivatives on catechol-O-methyl transferase activity in rat striatal tissue. RSC Adv..

[157] Calne S., Schoenberg B., Martin W., Uitti R.J., Spencer P., Calne D.B. (1987). Familial Parkinson’s disease: Possible role of environmental factors. The Canadian journal of neurological sciences. J. Can. Sci. Neurol..

[158] Schoenberg B.S. (1987). Environmental risk factors for Parkinson’s disease: The epidemiologic evidence. The Canadian journal of neurological sciences. J. Can. Sci. Neurol..

[159] Kitada T., Asakawa S., Hattori N., Matsumine H., Yamamura Y., Minoshima S., Yokochi M., Mizuno Y., Shimizu N. (1998). Mutations in the parkin gene cause autosomal recessive juvenile parkinsonism. Nature.

[160] Polymeropoulos M.H., Lavedan C., Leroy E., Ide S.E., Dehejia A., Dutra A., Pike B., Root H., Rubenstein J., Boyer R. (1997). Mutation in the α-synuclein gene identified in families with Parkinson’s disease. Science.

[161] Spillantini M.G., Schmidt M.L., Lee V.M., Trojanowski J.Q., Jakes R., Goedert M. (1997). α-synuclein in Lewy bodies. Nature.

[162] Valente E.M., Abou-Sleiman P.M., Caputo V., Muqit M.M., Harvey K., Gispert S., Ali Z., Del Turco D., Bentivoglio A.R., Healy D.G. (2004). Hereditary early-onset Parkinson’s disease caused by mutations in PINK1. Science.

[163] Klein C., Westenberger A. (2012). Genetics of Parkinson’s disease. Cold Spring Harb. Perspect. Med..

[164] Zucca F.A., Segura-Aguilar J., Ferrari E., Munoz P., Paris I., Sulzer D., Sarna T., Casella L., Zecca L. (2017). Interactions of iron, dopamine and neuromelanin pathways in brain aging and Parkinson’s disease. Prog. Neurobiol..

[165] Sulzer D., Zecca L. (2000). Intraneuronal dopamine-quinone synthesis: A review. Neurotox. Res..

[166] Segura-Aguilar J., Paris I., Muñoz P., Ferrari E., Zecca L., Zucca F.A. (2014). Protective and toxic roles of dopamine in Parkinson’s disease. J. Neurochem..

[167] Hastings T.G. (2009). The role of dopamine oxidation in mitochondrial dysfunction: Implications for Parkinson’s disease. J. Bioenerg. Biomembr..

[168] Dias V., Junn E., Mouradian M.M. (2013). The role of oxidative stress in Parkinson’s disease. J. Parkinsons Dis..

[169] Yuan H., Zhang Z.W., Liang L.W., Shen Q., Wang X.D., Ren S.M., Ma H.J., Jiao S.J., Liu P. (2010). Treatment strategies for Parkinson’s disease. Neurosci. Bull..

[170] Schapira A.H. (2011). Monoamine oxidase B inhibitors for the treatment of Parkinson’s disease: A review of symptomatic and potential disease-modifying effects. CNS Drugs.

[171] Connolly B.S., Lang A.E. (2014). Pharmacological treatment of Parkinson disease: A review. JAMA.

[172] Sarrafchi A., Bahmani M., Shirzad H., Rafieian-Kopaei M. (2016). Oxidative stress and Parkinson’s disease: New hopes in treatment with herbal antioxidants. Curr. Pharm. Des..

[173] Tarozzi A., Morroni F., Merlicco A., Hrelia S., Angeloni C., Giorgio C.-F., Hrelia P. (2009). Sulforaphane as an inducer of glutathione prevents oxidative stress-induced cell death in a dopaminergic-like neuroblastoma cell line. J. Neurochem..

[174] Khuwaja G., Khan M.M., Ishrat T., Ahmad A., Raza S.S., Ashafaq M., Javed H., Khan M.B., Khan A., Vaibhav K. (2011). Neuroprotective effects of curcumin on 6-hydroxydopamine-induced Parkinsonism in rats: Behavioral, neurochemical and immunohistochemical studies. Brain Res..

[175] Maher P. (2017). Protective effects of fisetin and other berry flavonoids in Parkinson’s disease. Food Funct..

[176] Dewapriya P., Himaya S.W., Li Y.X., Kim S.K. (2013). Tyrosol exerts a protective effect against dopaminergic neuronal cell death in in vitro model of Parkinson’s disease. Food Chem..

[177] Schapira A.H. (2008). Mitochondria in the aetiology and pathogenesis of Parkinson’s disease. Lancet Neurol..

[178] Pasban-Aliabadi H., Esmaeili-Mahani S., Sheibani V., Abbasnejad M., Mehdizadeh A., Yaghoobi M.M. (2013). Inhibition of 6-hydroxydopamine-induced PC12 cell apoptosis by olive (*Olea europaea* L.) leaf extract is performed by its main component oleuropein. Rejuvenation Res..

[179] Yu G., Deng A., Tang W., Ma J., Yuan C. (2016). Hydroxytyrosol induces phase II detoxifying enzyme expression and effectively protects dopaminergic cells against dopamine- and 6-hydroxydopamine induced cytotoxicity. Neurochem. Int..

[180] Spencer J.P., Whiteman M., Jenner P., Halliwell B. (2002). 5-s-Cysteinyl-conjugates of catecholamines induce cell damage, extensive DNA base modification and increases in caspase-3 activity in neurons. J. Neurochem..

[181] Goldstein D.S., Jinsmaa Y., Sullivan P., Holmes C., Kopin I.J., Sharabi Y. (2016). 3,4-Dihydroxyphenylethanol (Hydroxytyrosol) Mitigates the Increase in Spontaneous Oxidation of Dopamine During Monoamine Oxidase Inhibition in PC12 Cells. Neurochem. Res..

[182] Vauzour D., Corona G., Spencer J.P. (2010). Caffeic acid, tyrosol and p-coumaric acid are potent inhibitors of 5-S-cysteinyl-dopamine induced neurotoxicity. Arch. Biochem. Biophys..

[183] De Groot C.J., Bergers E., Kamphorst W., Ravid R., Polman C.H., Barkhof F., Van Der Valk P. (2001). Post-mortem MRI-guided sampling of multiple sclerosis brain lesions: Increased yield of active demyelinating and (p)reactive lesions. Brain.

[184] Luo C., Jian C., Liao Y., Huang Q., Wu Y., Liu X., Zou D., Wu Y. (2017). The role of microglia in multiple sclerosis. Neuropsychiatr. Dis. Treat..

[185] Browne P., Chandraratna D., Angood C., Tremlett H., Baker C., Taylor B.V., Thompson A.J. (2014). Atlas of Multiple Sclerosis 2013, A growing global problem with widespread inequity. Neurology.

[186] Sospedra M., Martin R. (2005). Immunology of multiple sclerosis. Annu. Rev. Immunol..

[187] Sospedra M., Martin R. (2016). Immunology of Multiple Sclerosis. Semin. Neurol..

[188] Gilgun-Sherki Y., Melamed E., Offen D. (2004). The role of oxidative stress in the pathogenesis of multiple sclerosis: The need for effective antioxidant therapy. J. Neurol..

[189] Pun P.B., Lu J., Moochhala S. (2009). Involvement of ROS in BBB dysfunction. Free Radic. Res..

[190] Aparicio-Soto M., Sánchez-Hidalgo M., Rosillo M., Castejón M.L., Alarcón-de-la-Lastra C. (2016). Extra virgin olive oil: A key functional food for prevention of immune-inflammatory diseases. Food Funct..

[191] Rosenberg G.A., Dencoff J.E., Correa N., Reiners M., Ford C.C. (1996). Effect of steroids on CSF matrix metalloproteinases in multiple sclerosis: Relation to blood-brain barrier injury. Neurology.

[192] Agrawal S.M., Lau L., Yong V.W. (2008). MMPs in the central nervous system: Where the good guys go bad. Semin. Cell. Dev. Biol..

[193] Liuzzi G.M., Latronico T., Branà M.T., Gramegna P., Coniglio M.G., Rossano R., Larocca M., Riccio P. (2011). Structure-dependent inhibition of gelatinases by dietary antioxidants in rat astrocytes and sera of multiple sclerosis patients. Neurochem. Res..

[194] Martín R., Hernández M., Córdova C., Nieto M.L. (2012). Natural triterpenes modulate immune-inflammatory markers of experimental autoimmune encephalomyelitis: Therapeutic implications for multiple sclerosis. Br. J. Pharmacol..

[195] Martín R., Carvalho-Tavares J., Hernández M., Arnés M., Ruiz-Gutiérrez V., Nieto M.L. (2010). Beneficial actions of oleanolic acid in an experimental model of multiple sclerosis: A potential therapeutic role. Biochem. Pharmacol..

[196] Riccio P., Rossano R., Liuzzi G.M. (2011). May diet and dietary supplements improve the wellness of multiple sclerosis patients? A molecular approach. Autoimmune Dis..

[197] Rowland L.P., Shneider N.A. (2001). Amyotrophic lateral sclerosis. N. Engl. J. Med..

[198] Cozzolino M., Ferri A., Carrì M.T. (2008). Amyotrophic lateral sclerosis: From current developments in the laboratory to clinical implications. Antioxid. Redox Signal..

[199] Boillée S., Vande Velde C., Cleveland D.W. (2006). ALS: A disease of motor neurons and their nonneuronal neighbors. Neuron.

[200] Kaur S.J., McKeown S.R., Rashid S. (2016). Mutant SOD1 mediated pathogenesis of Amyotrophic Lateral Sclerosis. Gene.

[201] Rizzardini M., Mangolini A., Lupi M., Ubezio P., Bendotti C., Cantoni L. (2005). Low levels of ALS-linked Cu/Zn superoxide dismutase increase the production of reactive oxygen species and cause mitochondrial damage and death in motor neuron-like cells. J. Neurol. Sci..

[202] Gurney M.E., Pu H., Chiu A.Y., Dal Canto M.C., Polchow C.Y., Alexander D.D., Caliendo J., Hentati A., Kwon Y.W., Deng H.X. (1994). Motor neuron degeneration in mice that express a human Cu,Zn superoxide dismutase mutation. Science.

[203] Zoccolella S., Santamato A., Lamberti P. (2009). Current and emerging treatments for amyotrophic lateral sclerosis. Neuropsychiatr. Dis. Treat..

[204] Cleveland D.W., Rothstein J.D. (2001). From Charcot to Lou Gehrig: Deciphering selective motor neuron death in ALS. Nat. Rev. Neurosci..

[205] McCombe P.A., Henderson R.D. (2011). The Role of immune and inflammatory mechanisms in ALS. Curr. Mol. Med..

[206] De Paola M., Sestito S.E., Mariani A., Memo C., Fanelli R., Freschi M., Bendotti C., Calabrese V., Peri F. (2016). Synthetic and natural small molecule TLR4 antagonists inhibit motoneuron death in cultures from ALS mouse model. Pharmacol. Res..

[207] Casula M., Iyer A.M., Spliet W.G., Anink J.J., Steentjes K., Sta M., Troost D., Aronica E. (2011). Toll-like receptor signaling in amyotrophic lateral sclerosis spinal cord tissue. Neuroscience.

[208] Berr C., Portet F., Carriere I., Akbaraly T.N., Feart C., Gourlet V., Combe N., Barberger-Gateau P., Ritchie K. (2009). Olive oil and cognition: Results from the three-city study. Dement. Geriatr. Cogn. Disord..

[209] Martínez-Lapiscina E.H., Clavero P., Toledo E., Estruch R., Salas-Salvadó J., San Julián B., Sanchez-Tainta A., Ros E., Valls-Pedret C., Martinez-Gonzalez M.Á. (2013). Mediterranean diet improves cognition: The PREDIMED-NAVARRA randomised trial. J. Neurol. Neurosurg. Psychiatry.

